# Cooperative Effect of Cations and Catalyst Structure in Tuning Alkaline Hydrogen Evolution on Pt Electrodes

**DOI:** 10.1021/jacs.3c11866

**Published:** 2024-03-07

**Authors:** Akansha Goyal, Sheena Louisia, Pricilla Moerland, Marc T. M. Koper

**Affiliations:** Leiden Institute of Chemistry, Leiden University, P.O. Box 9502, 2300 RA Leiden, The Netherlands

## Abstract

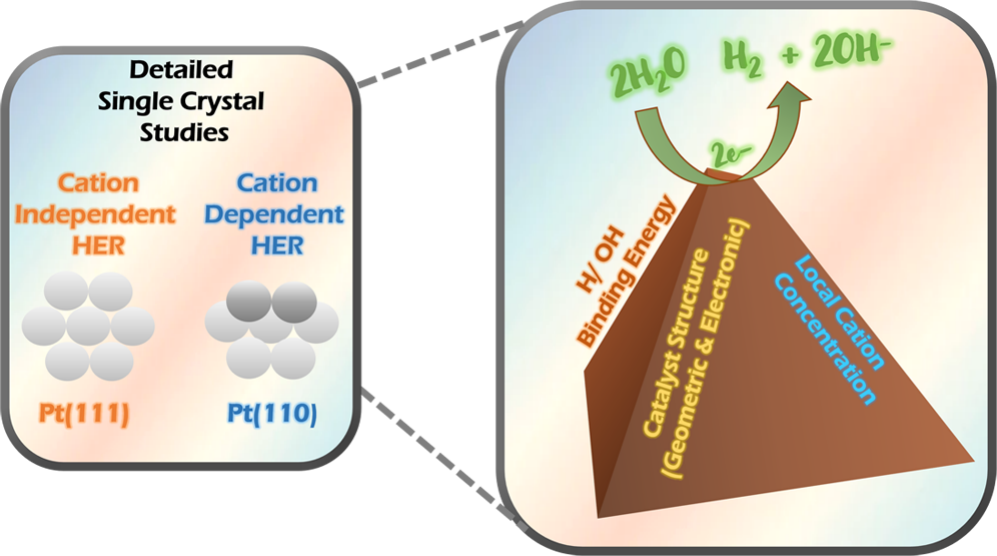

The kinetics of hydrogen evolution reaction (HER) in alkaline media, a reaction central to alkaline water electrolyzers, is not accurately captured by traditional adsorption-based activity descriptors. As a result, the exact mechanism and the main driving force for the water reduction or HER rate remain hotly debated. Here, we perform extensive kinetic measurements on the pH- and cation-dependent HER rate on Pt single-crystal electrodes in alkaline conditions. We find that cations interacting with Pt step sites control the HER activity, while they interact only weakly with Pt(111) and Pt(100) terraces and, therefore, cations do not affect HER kinetics on terrace sites. This is reflected by divergent activity trends as a function of pH as well as cation concentration on stepped Pt surfaces vs Pt surfaces that do not feature steps, such as Pt(111). We show that HER activity can be optimized by rationally tuning these step–cation interactions via selective adatom deposition at the steps and by choosing an optimal electrolyte composition. Our work shows that the catalyst and the electrolyte must be tailored in conjunction to achieve the highest possible HER activity.

## Introduction

Hydrogen evolution reaction (HER) has long served as a cornerstone in fundamental electrode kinetics, as many of the theories of electrocatalysis have been developed to explain the empirically observed activity trends for HER. This two-electron transfer reaction is also an important piece in the energy transition puzzle, as green hydrogen generated by water electrolysis is vital for achieving a carbon-neutral energy system and establishing a hydrogen economy.^1,2^

In this respect, alkaline water electrolyzers pose an advantage when compared to their acidic counterparts, as cheaper Ni-based catalysts show good HER and oxygen evolution reaction (OER) activity in alkaline media in comparison to expensive platinum and iridium, the best catalysts for HER and OER in acidic media, respectively.^3,4^ However, the energy efficiency of alkaline water electrolyzers remains inferior to proton exchange membrane (PEM) electrolyzers, as the HER activity is lower in the alkaline environment, regardless of the catalyst employed.^5,6^ As a result, significant research efforts have been made toward understanding the HER mechanism in alkaline media.

Various interfacial parameters have been shown or suggested to influence the HER activity in alkaline media, such as interfacial electric field strength,^7^ cation identity,^8−10^ oxophylicity of reactive sites,^11^ solvent reorganization energy,^12,13^ binding strength for hydroxyl ion, and cation–hydroxide interactions,^14−17^ to name a few. These parameters seem to influence activity differently, depending on the exact nature of the catalyst–electrolyte interface. As a consequence, several ambiguities still remain, as different groups have advocated different activity descriptors.^18,19^ Moreover, it is difficult to accurately map the poorly defined nature of Ni(OH)_2_-modified Pt catalysts as well as other metal hydroxide-modified surfaces onto a quantitative activity descriptor,^20^ adding further uncertainty to the problem.

To bypass these issues and to unravel the complex nature of the electrode–electrolyte interface during HER, here we systematically study electrolyte effects for HER on well-defined Pt surfaces, first by varying their geometric structure and then by selectively modifying them with adatoms. The key finding of this study is the demonstration of a fundamental difference between the cation–step site interaction and the cation–terrace interaction at Pt surfaces. With terraces, cations interact weakly and, as a result, do not influence the HER mechanism. By contrast, the cations interact strongly with Pt steps and play a crucial role in tuning the HER activity on stepped Pt surfaces. We then modify the oxophylicity of Pt steps via controlled adatom deposition to show that we can predictively alter the HER activity at the catalyst surface by tuning local oxophylicity in tandem with cation promotion effects. These results on well-defined Pt single crystals thereby paint a comprehensive molecular picture of what determines HER activity in alkaline media, providing a blueprint for HER optimization on more practical electrode geometries.

## Results and Discussion

**Figure 1:**
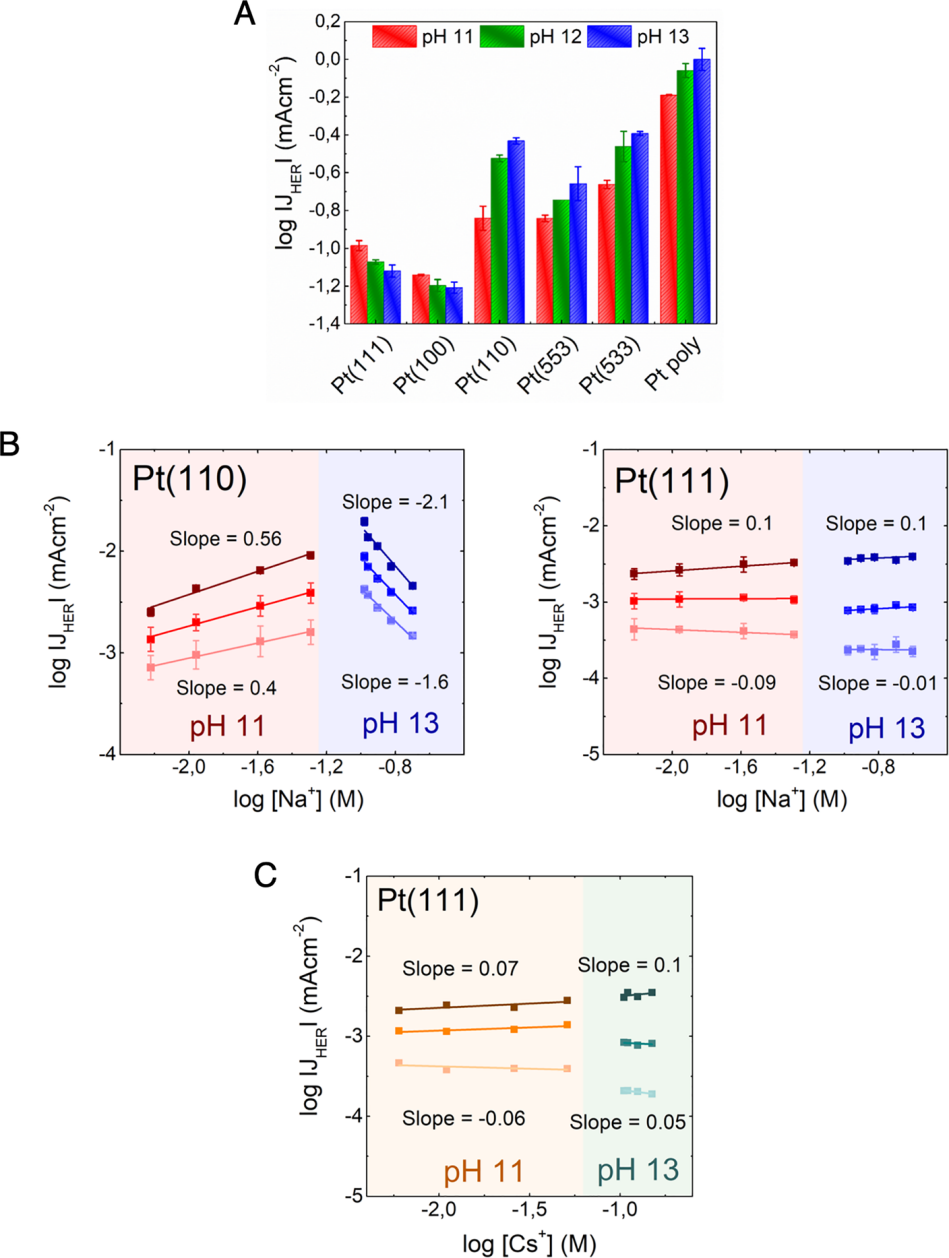
Electrolyte effects for HER on different Pt surfaces. (A) Activity for hydrogen evolution (log of current density at −0.05 V_RHE_ (*iR* corrected); here, a more positive value corresponds to higher activity) as a function of electrolyte pH (pH 11, pH 12, and pH 13) as recorded at different Pt surfaces. (B) Logarithm of HER current density plotted against the logarithm of [Na^+^] ion concentration at pH 11 (red) and pH 13 (blue) at 3 different potentials (vs RHE, *iR* corrected), – 0.01, – 0.05, and −0.1 V (light to dark) on Pt(110) and Pt(111). The corresponding slopes (reaction orders) are indicated next to the plots, where the slope at the bottom corresponds to the applied potential of −0.01 V_RHE_ (*iR* corrected) and the slope at the top corresponds to the applied potential of −0.1 V_RHE_ (*iR* corrected). Here, a positive slope indicates that increasing cation concentration enhances HER activity, while a negative slope indicates that increasing cation concentration leads to lower HER activity, and a reaction order close to zero indicates that cations do not affect HER. (C) Logarithm of HER current density plotted against the logarithm of [Cs^+^] ion concentration at pH 11 (orange) and pH 13 (cyan) as recorded at 3 different potentials (vs RHE, *iR* corrected), namely, −0.01, −0.05, −0.1 V (light to dark) on Pt(111). More detailed information on surface preparation, blank voltammograms, and measurements with other cations can be found in the Materials and Methods section and Supporting Information.^21−23^

First, the pH dependence of HER on different low-index Pt single crystals is established by varying the electrolyte pH from 11 to 13 while keeping the overall cation concentration constant at 0.1 M by using the NaClO_4_ salt. We note that ClO_4_^–^ anions exhibit negligible interaction with Pt(*hkl*) surfaces at HER relevant potentials;^24−26^ hence, any changes in the HER activity, as shown in Figure 1, cannot arise from ClO_4_^–^ anion adsorption at the surface. For Pt(111) and Pt(100), we observe a drop in HER activity with increasing electrolyte pH (Figure 1A; also see Figures S4 and S5), the decrease being more pronounced on Pt(111). By contrast, on Pt(110), we observe an enhancement in HER activity with increasing electrolyte pH (Figure 1A; also see Figures S4 and S5). Among these three low-index surfaces, Pt(110) differs from the other two, as it features step-type sites both in its unreconstructed and reconstructed form.^22,23,27^ To investigate whether the disparate pH trend on Pt(110) is due to the presence of steps on this surface, we studied the pH dependence of HER on Pt(553) and Pt(533). We chose these high-index stepped Pt surfaces as they differ in their step orientation but otherwise feature (111) terraces of similar width. The Pt(553) surface features steps of (110) orientation, whereas Pt(533) surface features steps of (100) orientation. Both Pt(553) and Pt(533) also show an enhancement in HER activity, similar to Pt(110), as the electrolyte pH is increased from 11 to 13 (Figure 1A; also see Figures S4 and S5). These results show that regardless of the step orientation, increasing pH results in increasing HER activity on stepped Pt surfaces, while on surfaces with mainly terraces, the opposite trend prevails. Similar to the stepped surfaces, polycrystalline Pt also shows an enhancement in HER activity with increasing pH (Figure 1A; also see Figures S4 and S5). This is not surprising since a polycrystalline surface is generally composed of randomly distributed crystallographic orientations and features various defects, i.e., steps and kink sites.^28^ Hence, the trend on polycrystalline Pt is expected to fall in line with stepped Pt surfaces. We note that a polycrystalline Pt surface not only comprises step sites but also other surface features (such as kinks, defects, and grain boundaries), which might interact more strongly with surface cations. Interestingly, some recent studies have also observed a similar pH trend on polycrystalline Pt and Pt nanoparticle catalysts,^10,29,30^ but no clear molecular picture has emerged thus far to explain why the trends on these catalysts differ from Pt(111).

To understand the molecular origin of these disparate pH trends, we probe the cation concentration effects on these surfaces. We expect these trends to be related because changes in the electrolyte pH invariably influence the near-surface cation concentration by tuning the interfacial electric field strength.^7^ In brief, at an electrode–electrolyte interface, the electric field is determined by the difference between the applied potential and the potential of zero charge (*pzc*) of the electrode material, i.e., Δ*E = E* – *E_pzc_*. The *pzc* is a material property of the electrode and represents the potential at which there is net zero (free) surface charge density at the electrode surface. Its value depends on both the electronic and geometric structure of the electrode. It is a constant on the NHE potential scale, but when the potential is referenced on the RHE scale, it depends on the solution pH, that is, the pzc shifts positively with 60 mV/pH on the RHE scale.^31,32^ Pt(111) is the only platinum surface for which a clear double-layer window exists, and so the only surface with a measurable *E_pzc_*, of 0.3 V_NHE_, which corresponds to >1 V_RHE_ at pH 11 and 13.^33,34^ Hence, at HER relevant potentials that are (much) more negative than the *pzc*, the electric field at the surface is negative and there is a net accumulation of cations near the electrode surface. The *pzc* shifts to a more positive potential on the RHE scale with increasing electrolyte pH and, consequently, the interfacial electric field (Δ*E = E* – *E_pzc_*) at a fixed potential on the RHE scale becomes more negative with increasing electrolyte pH.^35,36^ As a result, the near-surface cation concentration also increases with increasing electrolyte pH, and hence, these two electrolyte parameters are intimately interlinked with each other.^7^ Besides bulk pH and bulk cation concentration, the actual local current density also determines the near-surface cation concentration. We do not have a consistent model for this interplay yet.^7^

Figure 1B,C shows that Pt(111) and Pt(110) show not only a disparate dependence on the electrolyte pH but also on the cation concentration. Pt(100) behaves qualitatively similar to Pt(111), in agreement with the similar pH dependence, though experiments on Pt(100) are less reproducible as the preparation of Pt(100) typically leads to a number of defects that is difficult to control (see Supporting Information Figure S6). On Pt(110), increasing cation concentration results in increasing HER activity at pH 11, while at pH 13, increasing cation concentration results in an activity decrease (Figure 1B; also see Figure S7). The results on Pt(110) suggest that at a relatively low near-surface concentration (pH 11), cations assist in the HER activity, while at a high near-surface concentration (pH 13), cations inhibit HER (at pH = 11, Cs^+^ show a better promotional effect than Na^+^, as shown in Figure S7, but we note that the exact transition from promotional to inhibitive depends on multiple factors). These results are in good agreement with our previous observations on Pt and Au polycrystalline surfaces, where we observed a similar transition from a “promotional” regime at relatively low local cation concentration at pH 11 to an “inhibitive” regime at relatively high cation concentration at pH 13.^7,10^ In our previous work, we argued that in the promotional regime, cations assist in HER mechanism by stabilizing the dissociating water molecule at the interface (H_2_O + e^–^ + * + cat^+^ → *H – OH^δ−^···cat^+^ + (1 – δ)e^–^ → *H + OH^–^ + cat^+^). This also explains the increase in HER activity with increasing pH on stepped Pt surfaces because, as explained in the previous paragraph, an increasing electrolyte pH results in a higher near-surface cation concentration, thus enhancing the HER activity. However, beyond a threshold local concentration (at very high ionic strength and electrolyte pH), the cations appear to inhibit HER, and a negative reaction order in cation concentration is obtained. The exact reason for this inhibition of HER rate at high local concentration is currently not known. However, various possible explanations may be put forward. There could be site-blocking due to the chemical adsorption of cations at Pt steps.^37,38^ Another possibility could be the crowding of the double layer with cations,^39,40^ leaving less space for reactive water to interact with the electrode surface. Conversely, near-surface cations can also inhibit the HER activity by directly altering the structure of the interfacial water adlayer, as has been observed by Feliu et al. for hydrogen peroxide reduction reaction.^41^ Importantly, this duality of cation effects in tuning HER activity provides a possibility to rationally accelerate HER in commercial alkaline water electrolyzers by choosing an optimal electrolyte for a given catalyst. Elucidating this effect of local high cation concentration is clearly an important topic for future fundamental work.

By contrast, on Pt(111), cations do not affect the HER activity in either direction, i.e., on Pt(111), the HER activity remains nearly unchanged with increasing cation concentration, both at pH 11 and at pH 13 (Figure 1B; also see Figures S8 and S9). The absence of cation concentration effects for HER on the Pt(111) surface is also confirmed by a constant HER activity with increasing concentration of Cs^+^ ion in the electrolyte (Figure 1C; also see Figure S10). This is noteworthy because, among the different alkali metal cations, Cs^+^ ions have the strongest interaction with the Pt surface, owing to their low hydration energy.^38,39^ Hence, Cs^+^ ions should exert the strongest effect on the HER activity among all alkali metal cations (as also seen on Pt(110) in Figure S11). Clearly, these results show that there is an important distinction in the interaction between cations and Pt step sites compared to the interaction between cations and Pt terraces. Therefore, the atomic-level structure of the Pt catalyst determines whether and how cations participate in the HER mechanism.

**Figure 2:**
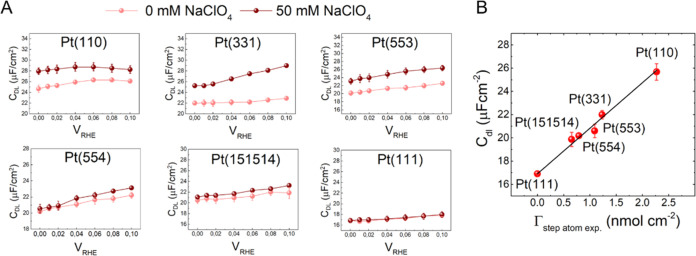
EIS measurements at different Pt single crystals. Specific double-layer capacitance (*C*_dl_; μF cm^–2^), as derived from the equivalent electric circuit (EEC) shown in Supporting Information Figure S15, on different Pt single crystals at pH 11: (A) with 0 mM NaClO_4_ (light red) and 50 mM NaClO_4_ (dark red) in the (vs RHE) in the H_upd_ region and (B) as a function of experimentally derived step density (Γ_step atom exp._; nmol cm^–2^) at the thermodynamic onset potential of HER, i.e., 0 V_RHE_ at pH 11 with 0 mM added NaClO_4_. The experimental step density (Γ_step atom exp._) was calculated by integrating the charge for the hydrogen adsorption peak as obtained from the blank cyclic voltammograms shown in Figure S12. Nyquist admittance plots, along with the impedance fits, are shown in Supporting Information Figures S14 and S15. *C*_dl_ as a function of Γ_step atom exp._ in the entire potential window is shown in Figures S16 and S18. In Supporting Information Figures S17 and S19, *C*_dl_ is plotted against the theoretically calculated step density (Γ_step atom theo._; where the (1 × 2) reconstruction of the surface is not considered).

To shed further light on the near-surface reaction environment at the different Pt surfaces, we performed capacitance measurements using electrochemical impedance spectroscopy (EIS) at different Pt single crystals with varying step densities (Γ_step atom_). We focus specifically on Pt surfaces that feature (111) terraces of variable length separated by (110) steps ((Pt(S)-[*n*(111) × (110)])), where Γ_step atom_ = 0 corresponds to Pt(111) (also see Supplementary text in the SI). Although we expect the effect of steps to also play an important role in the case of Pt(S)-[*n*(100) × (110)] surfaces, this work focuses on surfaces featuring (111) terraces. The interfacial capacitance gives information about the structure/composition of the electrical double layer, as variations in the double-layer capacitance (*C*_dl_) are directly correlated to the near-surface electrolyte concentration/composition (for a more detailed discussion, refer to the Supporting Text in the SI). We measure the double-layer capacitance at potentials 0.0–0.1 V vs RHE, where the Pt surface is covered with adsorbed hydrogen (H_ads_), as this is the relevant state of the surface for HER. We make use of the fact that in alkaline media, the formation of adsorbed hydrogen is kinetically slow, so that we can separate the *C*_dl_ from the adsorption (pseudo) capacitance. That is, we measure the capacity of H-covered Pt surface without pseudocapacitive contribution.^42−44^ In accordance with the HER activity trends, *C*_dl_ measurements also show a diverging trend on Pt(111) compared to the stepped Pt surfaces. Essentially, we see that on all of the surfaces, except Pt(111), the *C*_dl_ increases with increasing cation concentration in the electrolyte (Figure 2A). Moreover, at a given bulk cation concentration, the double-layer capacitance scales with the step density of the Pt surface (Figure 2B; also see Figures S16 and S17). Hence, in going from Pt(111) to Pt(110), the double-layer capacitance scales linearly with step density for a fixed bulk cation concentration and fixed bulk pH at the same applied potential (0 V_RHE_) (Figure 2B; also see Figures S16–S19). Moreover, the increase in the double-layer capacitance as a function of bulk cation concentration also scales with the step density (see Figure S20). These results show: (i) the interaction of cations at Pt terraces is weak, as reflected by the lack of cation concentration dependence of *C*_dl_ on Pt(111) and (ii) the fact that *C*_dl_ ∝ Γ_step atom_ (and Δ*C*_dl_ ∝ Γ_step atom_) shows that the extent to which the cations interact with the Pt surface is directly correlated to the presence of steps at the surface. We note that the double-layer capacitance of hydrogen-covered Pt is significantly lower than that of “clean” Pt, specifically Pt(111).^45^ The value of ca. 17 μF cm^–2^ for H-covered Pt(111) is close to the capacitance of CO-covered Pt(111).^46^ This low value suggests that the capacitance of H–Pt(111) is determined by a “gap capacitance” caused by a hydrophobic surface. Ultrahigh vacuum surface science experiments have indeed shown that on Pt terraces, a highly ordered two-dimensional (2-D) network of adsorbed water (hydrogen) is formed, lending a hydrophobic character to the surface. In contrast, Pt steps exhibit a one-dimensional (1-D) interaction with the adsorbed water, resulting in a completely different desorption/adsorption behavior at Pt steps.^46^ Therefore, the picture that the capacitance data in Figure 2 paint is that hydrogen adsorption renders the Pt(111) terraces hydrophobic, repelling water and solvated ions from the surface and that cations accumulate specifically near the step sites. This is also corroborated by previous DFT studies, where it has been shown that cations can interact with Pt steps, thus resulting in the non-Nernstian shift of step-associated peak in the so-called hydrogen region.^37^ It is plausible that the strong interaction of cations at the steps is the result of the differences in the local electric field intensity near steps as compared to the terraces. In relation to this, we emphasize that we consider cations to accelerate the water dissociation reaction but not to affect or enhance OH adsorption in the HER-relevant potential window (as has been suggested recently).^16^ In fact, our previous work has shown that there is no clear evidence for OH adsorption on platinum single-crystal electrodes at potentials negative of the step-related peaks; for a detailed discussion, see ref (47).

**Figure 3:**
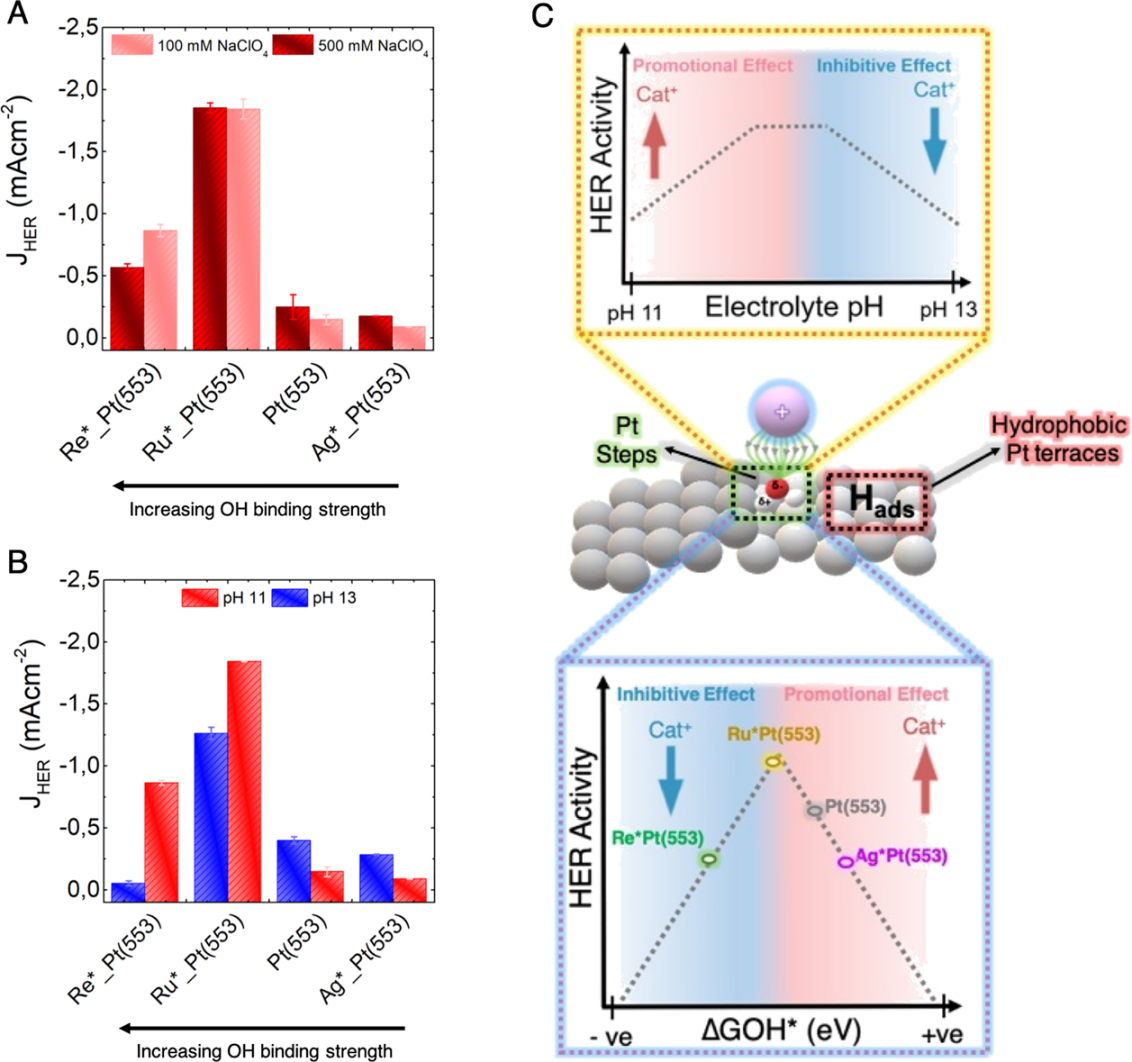
HER activity trends on different adatom modified Pt(553) surfaces. HER current density (mA/cm^2^) at −0.05 V_RHE_ (*iR* corrected) on different adatom modified Pt(553) surfaces and Pt(553) as a function of (A) Na^+^ ion concentration (100 mM and 500 mM NaClO_4_) in the electrolyte at pH 11 and (B) bulk electrolyte pH (pH 11 and pH 13). The blank cyclic voltammograms and HER cyclic voltammograms on different adatom modified surfaces are shown in Supporting Information Figures S21–S25. (C) Schematic representation of how different factors, including the bulk electrolyte pH, atomic structure of the catalyst surface, and the oxophylicity of the active step sites, all determine the overall rate of HER in alkaline media by tuning the local cation–step interactions.

The important consequence of this picture is that these differences in the near-surface reaction environment also lead to a mechanistic distinction for HER on Pt terraces vs steps. Namely, the cations act as “co-catalyst” on Pt steps, while they have an inconsequential role near Pt terraces (Figure 3C). As a result, electrolyte effects, including pH effects and cation concentration effects, play out differently on these two types of surface features. This also explains the discrepancies that exist in the literature where electrolyte effects have been studied on less well-defined catalysts, and, as a result, varying pH trends have been obtained for HER activity.^29,48−50^ Our results also confirm that electrolyte pH and near-surface cation concentration are two interrelated parameters and that it is not possible to change the former without changing the latter. Moreover, we note that while the interaction of steps and substrate molecules has long been regarded as an important reaction descriptor in electrocatalysis, our work shows that there is a need to expand this view to include step–cation interactions. These interactions are crucial for tuning the activity of catalysts for HER in alkaline media and possibly for other electrocatalytic reactions as well, especially reduction/hydrogenation reactions, which generally take place at potentials more negative of the *pzc* of a catalyst surface that is hydrogen-covered.

To demonstrate the generality of this insight, we also studied the role of near-surface cation concentration in tuning the HER activity on adatom modified stepped Pt(553) surfaces. It has been shown previously that certain adatoms can be selectively deposited on the step edges of Pt(553)^11,51^ and that the oxophylicity of the deposited adatoms tunes the HER activity.^11^ This is because oxophilic adatoms bind more strongly with the oxygen of the dissociating water molecule, thereby lowering the energy barrier for the rate-determining Volmer step. However, similar to the hydrogen-binding energy in acidic media,^4^ there is a volcano-type relationship between adatom oxophylicity and HER activity.^11^ Hence, a less oxophilic metal like Ag that binds hydroxyl ions more weakly than Pt (Δ*G*_OH*_^Ag^ = 0.2 eV at 0 V_RHE_) lies on the side of the activity volcano where oxygen binding is weak. On the other hand, a strongly oxophilic metal like Re (Δ*G*_OH*_^Re^ = −0.9 eV at 0 V_RHE_) lies on the strong binding leg of the volcano. Among the different surfaces studied, Ru-modified Pt(553) showed the highest HER activity, as its OH binding energy (Δ*G*_OH*_^Ru^ = −0.4 eV at 0 V_RHE_) is closest to the top of the volcano.^11^ To expand this understanding, here we studied electrolyte effects on Ag, Ru, and Re-modified Pt(553) surfaces. These adatom modified Pt(553) surfaces can essentially act as model catalysts to understand the cation–step interactions since they feature step sites of variable oxophilicity while maintaining a constant hydrogen-binding strength (on the terrace). The idea is to see whether cation–step interactions tune the HER activity on these modified catalysts in a consistent way. Indeed, as expected, on Ag-modified Pt(553), which interacts with water weakly, increasing the near-surface cation concentration, both by increasing the NaClO_4_ concentration in the electrolyte (at pH 11) and by increasing the electrolyte pH (11 to 13), enhances the HER activity (Figure 3A,B; also see Figures S24 and S25). On the other hand, on Re-modified Pt(553), the opposite trends are observed (Figure 3A,B; also see Figures S24 and S25). The trends on Ru-modified Pt(553) are somewhat intermediate compared to the other two surfaces (Figure 3A,B; also see Figures S24 and S25). It is important to note that on Ru-modified Pt(553), an increasing cation concentration at pH 11 seems to have no effect on HER activity at −0.05 V_RHE_. However, a closer look shows that at more negative overpotentials (Figure S24), HER activity decreases with increasing cation concentration on Ru-modified Pt(553). This is also in line with the more subdued inhibitive effect of electrolyte pH on this surface. These trends reaffirm that cations near the surface have a stabilizing effect for the dissociating water molecule at the interface (*H···OH^δ−^···cat^+^), as long as the interaction between steps and the substrate water molecules is not too strong (like on Ag-modified Pt(553)). Conversely, when the step-water interaction is too strong already (like on Re-modified Pt(553)), the barrier for the water dissociation step is already very low, and hence, the cations near the surface apparently have an inhibitive effect on HER activity (Figure 3C). In line with this, on Re- and Ru-modified Pt(553), the inhibitive effect of cations is already observed at pH 11, while on bare Pt(110), an inhibitive effect of cations is only observed at higher electrolyte pH and higher ionic strength (Figure 1B). Interestingly, the inhibitive effect of cations for HER activity is already observed at pH 11 on polycrystalline Pt electrodes.^10^ This indicates that in addition to the strength of the cation–step interactions, the population and type of defect (i.e., step and kink) sites at the catalyst surface can also determine the role of cations in tuning the HER activity. Future work will need to address the exact role of interfacial cations in this inhibitive regime, including their interaction with oxophilic surface modifiers such as Ru and Re.

**Figure 4:**
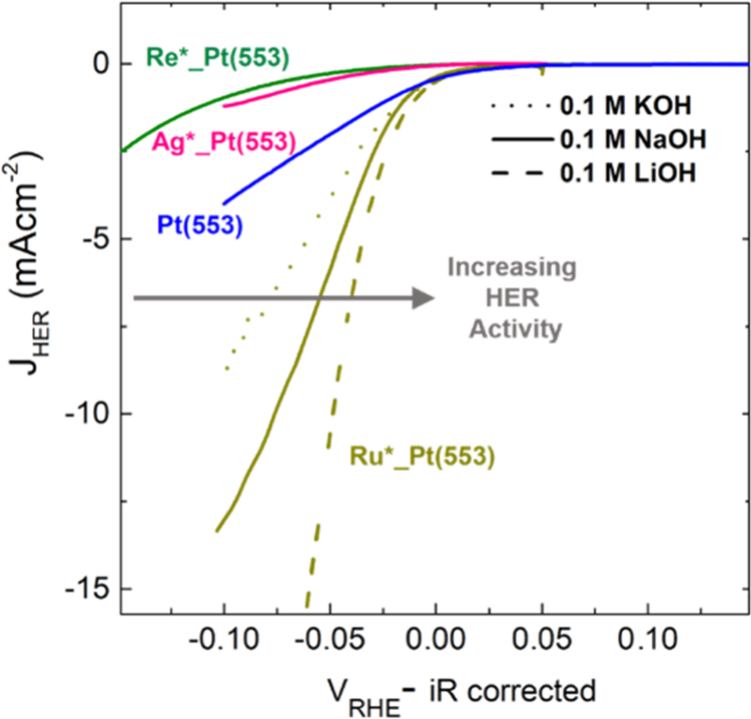
HER activity trends on Ru-modified Pt(553) in different alkali metal cation containing electrolytes: Cyclic voltammetry of Re*_Pt(553) (green), Ag*_Pt(553) (pink), Pt(553) (blue), and Ru*_Pt(553) (dark yellow) in 0.1 M KOH (dotted line), 0.1 M NaOH (solid line), and 0.1 M LiOH (dashed line).

These results on modified Pt catalysts highlight that for every catalyst, there is an optimal electrolyte composition that leads to the most favorable cation–catalyst interactions to yield the highest possible HER activity. To verify this conclusion, in Figure 4, we compare the activity of Ru-modified Pt(553) in different alkali metal containing electrolytes. The idea is to see whether the activity of this most optimal HER catalyst can be further improved by rationally tuning the identity of the electrolyte. As expected, among the different adatom modified surfaces, Ru-modified Pt(553) shows the highest HER activity. Remarkably, in going from KOH to LiOH, a further 3-fold increase in HER current density (at −0.05 V_RHE_) is observed for Ru-modified Pt(553). This activity enhancement in Li^+^ cation containing electrolyte is in line with our hypothesis that on a strongly oxophilic surface like Ru-modified Pt(553) cation–step interactions show an inhibitive effect for HER (Figure 3C). Consequently, a strongly hydrated cation like Li^+^ that interacts weakly with the catalyst surface^38,39^ exhibits the highest HER activity on Ru-modified Pt(553). Hence, depending on the oxophylicity of the catalyst surface, varying cation identity trends can be observed for HER. These results further emphasize that to improve the energy efficiency of HER in alkaline media, it is imperative to optimize the electrode and the electrolyte cooperatively.

## Conclusions

The results presented in this paper give a self-consistent picture of HER activity on Pt surfaces in alkaline media that emphasizes the key role of local cation–step interactions in activating water. We deliberately use the word “interaction” and not “adsorption” in relation to cations as we do not have detailed information about the exact nature of the cation interaction, whether they actually “contact adsorb” with the surface or not, and to what extent these “interfacial” cations should be considered discharged or not. Nevertheless, they clearly control interfacial reactivity to an important extent. Our paper ties together geometric effects (single crystals), electronic effects (via adatom tuning), and electrolyte effects (with respect to the pH, cation concentration and even the cation identity) into one single model that makes specific qualitative predictions about the most optimized electrode–electrolyte combination for alkaline HER. For instance, it shows that a highly active HER catalyst should run in Li-containing electrolyte and shows how this is the result of the subtle balance between optimizing (at least) three activity descriptors. The role of cations is comparable to that of oxophilic adatom modification but not identical: cations stabilize the transition state of the water dissociation reaction without promoting OH adsorption. However, we emphasize that the exact effect of the combination of electrocatalyst oxophilicity, pH, cation concentration, and cation identity is not a simple projection onto a single activity measurement. Our model offers guidelines rather than exact or quantitative activity predictions. Also, we would like to stress that H-free Pt(111) is probably the least suitable model surface for a real (polycrystalline) Pt electrode under HER conditions, and therefore, we strongly discourage the use of Pt(111) for model DFT calculations. The (111) terrace generally has a low activity for HER and a low tunability as cation promotors do not interact strongly enough with this facet, likely related to the hydrophobic character of the H–Pt(111) surface (Figure 3C). On the other hand, step and defect sites specifically accumulate cations and are thereby sensitive to cation promotion (either by increasing pH or higher bulk cation concentration). The remarkable conclusion is that steps enhance or influence HER not because they provide optimal binding energy for the catalytic intermediate but because they are the sites where the cations accumulate. If the step site has low oxophylicity, cations help the activation of water and increase HER activity. If step sites have high oxophylicity, the promotional role of cations is unhelpful to the extent that increasing their local concentration inhibits HER activity (Figure 3C). Therefore, the local cation–step interaction controls HER activity in (neutral and) alkaline media and requires careful optimization for each HER catalyst.

## Data Availability

All data are available in the main text or the Supporting Information.

## Supporting Information Available

A detailed Materials and Methods section describing the electrochemical experiments. Additional voltammetry, impedance spectroscopy, and capacitance data, as well as Tafel plots (Supporting Figures S1–S25, Table S1, Supporting Text and Materials and Methods) (PDF)
