# Supplementary material for: Cooperative Effect of Cations and Catalyst Structure in Tuning Alkaline Hydrogen Evolution on Pt Electrodes
Source: J Am Chem Soc. 2024 Mar 7;146(11):7305–12. doi: 10.1021/jacs.3c11866 (PMC10958517; doi:10.1021/jacs.3c11866)
Supplement: Supplementary file 1 — ja3c11866_si_001.pdf [file ja3c11866_si_001.pdf]

Supplementary Materials for

Cooperative Effect of Cations and Catalyst Structure in Tuning Alkaline Hydrogen Evolution on Pt electrodes

Akansha Goyal[1], Sheena Louisia[1], Pricilla Moerland[1], Marc T.M. Koper[1*]

Correspondence to: m.koper@chem.leidenuniv.nl

Materials and Methods

Chemicals and Materials

The electrolytes for blank cyclic voltammetry (CV) and HER activity measurements were prepared from H_2SO_4 (98% by wt. solution, EMSURE, Merck), $HClO_4$ (60% by wt. solution, EMSURE, Merck), $NaClO_4$ (99.99%, trace metals basis, Sigma-Aldrich), NaOH (32% by wt. solution, analysis grade, Merck), $CsClO_4$ (99.995%, Thermo-Scientific), CsOH (99.95%, Merck) and Ultrapure water (MilliQ gradient, ≥18.2 MΩcm, TOC < 5 ppb). Ar (6.0 purity, Linde) and H_2 (5.0 purity, Linde) were used for purging the electrolytes. The electrolytes for adatom electrodeposition were prepared from $AgClO_4$ (97%, Sigma-Aldrich), $RuCl_3.xH_2O$ (Aldrich Chemie) and NH_4ReO_4 (Johnson Matthey). Cut and polished disk type or bead type Pt single crystals of desired orientation were purchased from either MaTeck or Icryst.

Single Crystal Preparation

Prior to each experiment, the surface of a given Pt single crystal was first prepared by flame annealing (30 sec for a disk and 10 sec for a bead) and subsequent cooling (ca. 120 sec) in 1:2 Ar/CO atmosphere. Then the crystal was transferred to the working cell with a protective water droplet. CO atmosphere was used for cooling the Pt single crystals for two reasons: (i) strongly adsorbing gas like CO forms a protective adlayer at the surface which prevents any surface contamination or unwanted surface roughening due to ambient oxygen (ii) it has been shown previously that Pt single crystals prepared in CO atmosphere generally give well-ordered and unreconstructed surfaces.(*1-3*) More specifically, it has been shown both by Attard et al. and by Kolb et al. that CO atmosphere leads to the formation of unreconstructed Pt(110)-(1x1) surface.(*1, 3*) Moreover, Kolb et al. have shown both with *in-situ* STM measurements as well as with electrochemical measurements that only the Pt(100) surface prepared under CO atmosphere forms a flat, unreconstructed surface with large terraces free of islands or holes. In all other atmospheres, Pt(100) forms the hexagonally reconstructed structure with islands and defects.(*1*)
Prior to each measurement, the CO adlayer was electro-oxidatively desorbed from the Pt single crystal and thereafter the blank CVs and HER CVs were recorded. We note that based on the integrated charge of the CO stripping peak (Fig. S2), the total amount of CO_2 formed is quite small (ca. 10^{-12}-10^{-13} g_{CO_2}) and hence, we do not expect any concomitant carbonate contamination to affect our activity measurements, especially under HER relevant conditions.
For calculating the current densities, the geometric surface area of the respective single crystal was used.

Polycrystalline Pt Preparation

Before each experiment, Pt polycrystalline disk (diameter = 5mm, Pine instruments) was mechanically polished on Buehler micro-polishing cloth (8 inches) with decreasing sizes of diamond polishing suspension, namely, 3µm, 1 µm and 0.25µm. Next, the disk was sonicated

in ultrapure water and acetone for ca. 10 min. to remove any inorganic/organic impurities and mounted on the RDE tip (E6/E5 ChangeDisk tips in a PEEK shroud; Pine Research). Thereafter, the surface was electrochemically polished in 0.1 M H_2SO_4 electrolyte by cycling between 1.8 V_{RHE} to -0.5 V_{RHE} for 200 cycles at a scan rate of 1 Vs^{-1}. The representative CV of polycrystalline Pt was recorded by cycling the electrode in the same electrolyte between 0.05 V_{RHE} to 1.2 V_{RHE} at a scan rate of 50 mVs^{-1}. In order to calculate the current densities, the electrochemically active surface area (ECSA) of the electrode was determined by calculating the charge under the hydrogen desorption peak and dividing it by the specific charge associated with the oxidation of a monolayer of adsorbed hydrogen on Pt ($Q^{H}_{ox,ML,Pt}$ = 230 μCcm^{-2}).(*4*)

Electrochemical Measurements

The electrochemical measurements in alkaline media were carried out in a home-made PTFE cell while the measurements in acidic media were conducted in a homemade borosilicate glass cell. The reference electrode was separated from the working compartment with the help of a Luggin capillary and the counter electrode was a Pt wire (99.99% purity), unless otherwise stated. The components of the electrochemical cell were cleaned prior to each experiment by boiling them five times in ultrapure water. When not in use, they were stored in 1 g/L solution of $KMnO_4$. Before boiling, any traces of $KMnO_4$ and MnO_2 were removed by submerging them in a diluted solution of acidified H_2O_2 (few drops of conc. H_2SO_4 and 10-15 mL H_2O_2 in excess water) for half an hour. Before every experiment, the electrolytes were purged for ca. 30 min. with Ar to remove any dissolved oxygen form the electrolyte. Moreover, during the measurement, Ar was bubbled over the headspace of the electrochemical cell, in order to eliminate any interference from the ambient oxygen. A homemade reversible hydrogen electrode (RHE) was used as the reference electrode in all the experiments. All the electrochemical measurements were carried out using a Biologic (VSP-300) potentiostat. For all the measurements done with Pt single crystals a hanging meniscus configuration was used and for the measurements done with polycrystalline Pt, an MSR rotator (Pine Research) was used which was rotated at 2500 rpm. For all the CVs taken, 85% Ohmic drop compensation was performed and for all the steady-state potentiostatic measurements 100% Ohmic drop compensation was applied. Before measuring the HER activity, the blank CV of all the Pt surfaces were recorded (scan rate: 10 mV s^{-1}) in the working cell to check the quality of surface preparation. All the studies for the pH dependence of HER were done in Ar sat. 0.1 M electrolytes (xM NaOH + yM $NaClO_4$ such that x+y = 0.1). All the studies for the bulk cation concentration dependence of HER were done in Ar sat. electrolytes either at pH 11 (10^{-3} M NaOH/CsOH) or at pH 13 (0.1 M NaOH/CsOH) where the cation concentration was varied by adjusting the concentration of $NaClO_4$ or $CsClO_4$ in the electrolyte, such that the total concentration of perchlorate anions was similar at different pHs. The CVs for HER activity were taken in the potential window of 0 V_{RHE} to -0.1 V_{RHE} (iR corrected) at a scan rate of 10 mVs^{-1}. Additionally, to obtain the Tafel data, chronoamperometry was performed in the potential window of the CVs (0 V_{RHE} to -0.1 V_{RHE}; iR corrected) at 10mV potential steps. Generally, 20 seconds per potential step were enough to reach the steady state. EIS measurements were performed at pH 11 (10^{-3} M NaOH) either with 0 mM $NaClO_4$ or with 50 mM $NaClO_4$ containing electrolytes. The electrochemical impedance spectroscopy (EIS) measurements were done in the potential window of 0.1 V_{RHE} to 0 V_{RHE} at 20 mV steps, with frequencies ranging from 30 KHz to 1 Hz and a peak to peak amplitude of 5 mV. Moreover, a 10 µF shunt capacitor bridge was added between a secondary counter electrode (Pt wire) and the reference electrode in order to eliminate any artefacts caused by the non-ideal behavior of the potentiostat at high frequencies.(*5*) The impedance data was fit with an appropriate equivalent electrochemical circuit (EEC; shown in Fig. S14 or Fig. S15) with the help of EIS Zfit (part of Biologic's EC-Lab software).

Adatom Electrodeposition at Pt(553) Steps

Metal adatoms were selectively electrodeposited on Pt(553) steps by following the procedure outlined previously.(*6, 7*) Detailed characterization of these surfaces can be found there, and in Figs. S21-25. In brief, the as-prepared Pt(553) single crystal was transferred to an electrochemical cell containing 0.1 M $HClO_4$ plus 10^{-6} M salt of the respective adatom (i.e. $AgClO_4$ or $RuCl_3$ or NH_4ReO_4) and cycled for 10-200 cycles between 0.05 V_{RHE} to 0.35 V_{RHE}. Afterwards, the adatom modified Pt(553) surface was ready for electrochemical measurements (as outlined in the last subsection). To remove the adatoms from the surface, the electrode was subjected to repeated cycles of chemical etching (in conc. HNO_3) and flame annealing.

The adatom coverage at the step edge of Pt(553) was calculated by measuring the change in charge under the step-associated peak of Pt(553) in the blank cyclic voltammograms (0.05 V_{RHE} to 0.35 V_{RHE} on Re* Pt(553) and 0.05 V_{RHE} to 0.8V_{RHE} on all the other surfaces; see Fig. S21-23). For Re* Pt(553), potentials more anodic than 0.35 V_{RHE} were avoided to prevent oxidation of the adatoms. For correctly comparing the HER activity of different adatom modified surfaces, the current densities obtained for HER were normalized for the adatom coverage at the surface.(*6*)

Supplementary Text

Step Density Determination

The theoretical step density ($\Gamma_{\text{step atom theo.}}$) of Pt(S)-[n(111) x (110)] surfaces, namely, Pt(111), Pt(15,15,14), Pt(554), Pt(553), Pt(331) and Pt(110) can be calculated as $\Gamma_{\text{step atom theo.}} = 1/(n-2/3)$;(*8*) where n is the width of the terrace. In theory, the width of Pt(111) terrace is infinite which is reduced to 30 atoms in Pt(151514), 10 atoms in Pt(554), five atoms in Pt(553), 3 atoms in Pt(331) and 0 atoms in Pt(110). However, in practice, Pt(110) shows (1x2) missing row reconstruction which leads to the formation of two atom long (111) terraces and reduces its step density by half. Hence, the real step density of a surface can differ from the theoretical value, either due to the presence of any imperfections at the surface or due to reconstruction (such as in Pt(110)). That is why it is better to use the experimental step density ($\Gamma_{\text{step atom exp.}}$) or the surface concentration of step atoms (nmol cm^{-2}). The experimental step density of a surface can be estimated from the experimentally obtained charge for hydrogen desorption peak ($Q^H_{\text{ox,Pt(S)-[n(111) x (110)]}}$) as follows: $\Gamma_{\text{step atom exp.}} = \frac{Q^H_{\text{ox,Pt(S)}-[n(111)\,x\,(110)]}}{A.F}$; where F is Faraday's constant and A is the geometric area of the electrode.(*9*)

EIS data fitting and interpretation

The fitting of impedance data in the H_{upd} region is generally done by using the EEC shown in Fig. S14 (EEC1).(*10, 11*) In this case, only the adsorption of hydrogen is considered at the interface, and its kinetics is captured by R_{ad} and C_{ad}. In acidic media, the kinetics of $H_{adsorption}$ becomes too fast to measure (i.e. $R_{ad} \approx 0$) and this circuit reduces to a simple RC circuit where no real distinction can be made between the double layer capacitance (C_{dl}) and the pseudo-capacitance due to hydrogen adsorption (C_{ad}). However, in alkaline media, the kinetics of $H_{adsorption}$ is quite slow and consequently, double layer charging and $H_{adsorption}$ have two distinct time constants, such that C_{dl} and C_{ad} can be reliably separated from one another. We note that under ideal conditions, *i.e.* in the absence of any specific adsorption, the double layer capacitance (C_{dl}) is further represented by two capacitances that are in series with each other, C_i, the inner layer capacitance, and C_{diff}, the diffuse layer capacitance $\left(\frac{1}{C_{dl}} = \frac{1}{C_i} + \frac{1}{C_{diff}}\right)$, where at any point, C_{dl} is dominated by the smaller of the two capacitances.(*12*) According to Gouy-Chapman-Stern theory, C_i represents the capacitance due to the charged species that are located

in the Outer Helmholtz plane and should be independent of the electrolyte. On the other hand, C_{diff} represents the capacitance due to any charged species that are truly diffuse and it varies both with potential and with the identity/composition of the electrolyte. In theory, in dilute enough electrolytes, C_{diff} will dominate the overall double layer capacitance and it will be directly proportional to the square root of electrolyte concentration in the diffuse layer ($C_{diff} \propto \sqrt{Conc._{diff}}$). However, a recent study by our group has shown that even in very dilute electrolytes C_{dl} differs from the theoretical values expected from Gouy-Chapman theory ($C_{G\text{-}C}$).(*13*) This is due to the presence of attractive ion-surface interactions, which are not accounted for in the original GCS theory. Hence, even though some proportionality is expected between the C_{dl} and the electrolyte concentration in the diffuse layer, it will be more complicated than what is suggested by the simplified formula for C_{diff}. Nevertheless, based on the C_{dl} values we obtain by using EEC1 (Table S1, Fig. S14-S17), we can see that C_{dl} and hence the near surface concentration of cations clearly increases with increasing step density of Pt.

However, while the data at high frequencies shows a good fit with EEC1, at low frequencies a good fit is not obtained with EEC1, especially on stepped Pt single crystals (Fig. S14b). We note that because C_{dl} is mainly obtained due to the perturbations at high frequency (due to its small time constant), the poor quality of the fit at low frequencies should not have an impact on the correct estimation of the C_{dl} values. Nevertheless, we will briefly discuss what can be the possible reason for these discrepancies and what can be done to rectify this. One possible reason can be the co-adsorption of other species during the H_{upd} process. It is well-known that the step-associated peak on Pt arises due to the replacement of $H_{adsorbed}$ with $OH_{adsorbed}$.(*14*) Hence, in this potential range, co-adsorption of H and OH has to be considered, and a parallel arm has to be added to EEC1 to account for the kinetics of OH adsorption, as shown in Fig. S15 (EEC2). Moreover, this view has been further refined by our group recently, where we showed that alkali metal cations also co-adsorb along the steps and weaken OH adsorption, thus resulting in the non-Nernstian shift of this step associated peak. In view of this, perhaps an additional arm would be required to truly fit the impedance data around the potential window of the step-associated peak. We note that in our EIS measurements we stay at potentials more negative than the step-associated peak. Nevertheless, it has been shown with DFT calculations that in alkaline electrolytes (pH 14), cation co-adsorption is favorable on Pt single crystals, at potentials as negative as 0 V_{RHE}.(*15, 16*) Hence, the fact that our EIS data fits better with EEC2 (Fig. S15b) could suggest that there is in-fact cation specific adsorption taking place at the surface which competes with H adsorption. This can have interesting implications both with regards to the true surface composition of Pt during HER and with regards to exact nature in which the cations interact with dissociating water molecules at the interface. However, a detailed analysis of this requires a separate study unto itself and is therefore a topic of future work. Importantly, the value of C_{dl} obtained from the fits, remains largely unchanged regardless of the circuit employed (Table S1). This is understandable since the different circuits only account for the changes in the low frequency window of the measurements i.e. changes which are related to the adsorption phenomenon at the interface, while C_{dl}, as previously mentioned, presents itself in at higher frequencies. Regardless, in our paper we use EEC2 to plot the C_{dl} values as this circuit gives better overall fits for the EIS data.

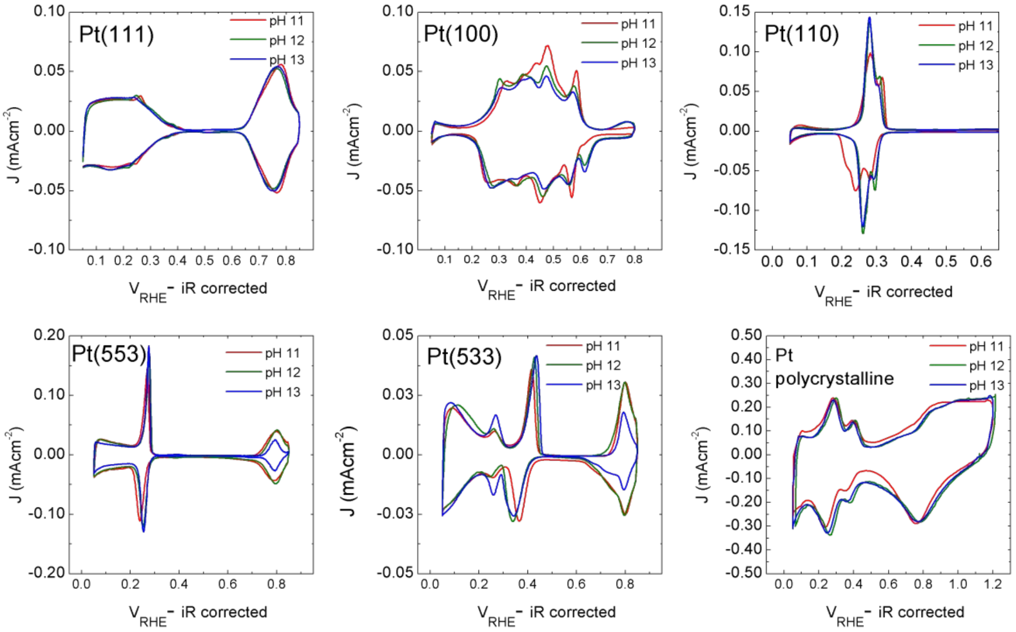

Fig. S1. *Blank CVs of Pt(111), Pt(100), Pt(110), Pt(553), Pt(533) and Pt polycrystalline as recorded in pH 11, pH 12 and pH 13 electrolytes at a scan rate of 10 mVs^{-1}. For the preparation of single crystals, the procedure outlined in Materials and Methods subsection 2 was followed and for the preparation of polycrystalline Pt the procedure outlined in Materials and Methods subsection 3 was followed.*

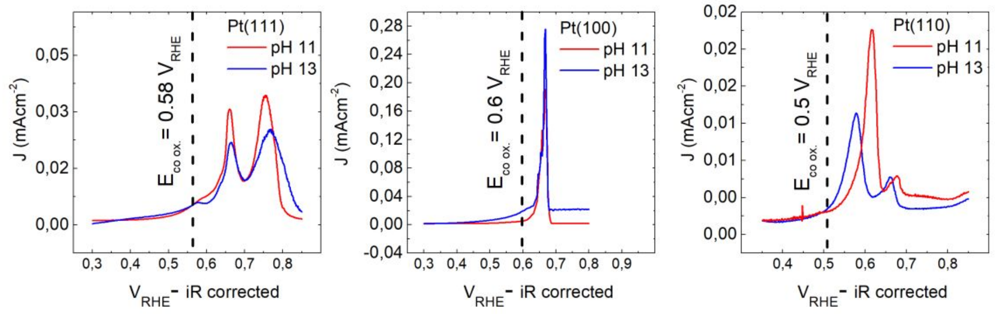

Fig. S2. *Electro-oxidative stripping of CO adlayer on Pt(111), Pt(100) and Pt(110) at pH 11 and pH 13 at a scan rate of 10 mV s^{-1}. Here the amount of CO oxidized (charge under the peak) can vary from one experiment to the other due to variations in transfer time from the cooling cell to the working cell. However, the onset potential of CO ox. (as indicated in each graph) can give information about the pH dependence and structure dependence of CO oxidation and therefore OH binding at the surface.(17)*

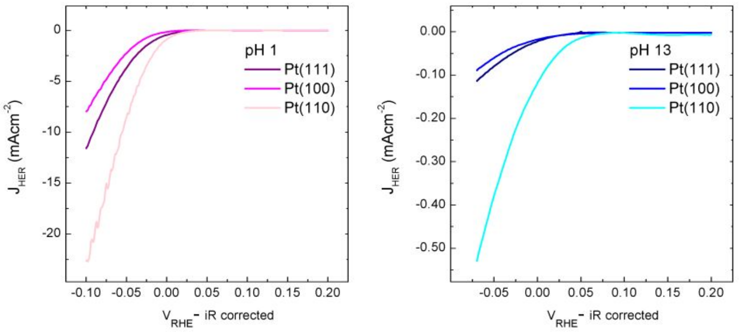

Fig. S3. *HER CVs of 3 basal Pt single crystals at pH 1 (0.1 M HClO4; left) and pH 13 (0.1 M NaOH; right) recorded at 10 mVs*$^{-1}$.

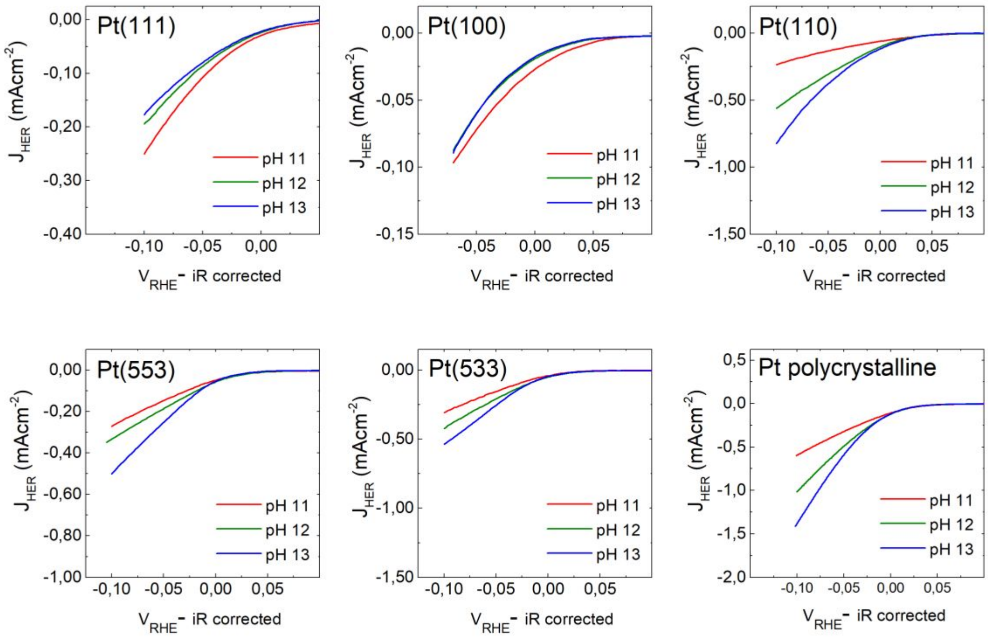

Fig. S4. *HER CVs at different Pt surfaces in 0.1 M NaOH (pH 13), 0.01 M NaOH +0.09 M NaClO4 (pH 12) and 0.001 M NaOH + 0.099 M NaClO4 (pH 11) recorded at 10 mVs*$^{-1}$. *All the measurements on Pt single crystals were done in stationary hanging meniscus configuration and the measurement at polycrystalline Pt rotating disk electrode was done at 2500 rpm.*

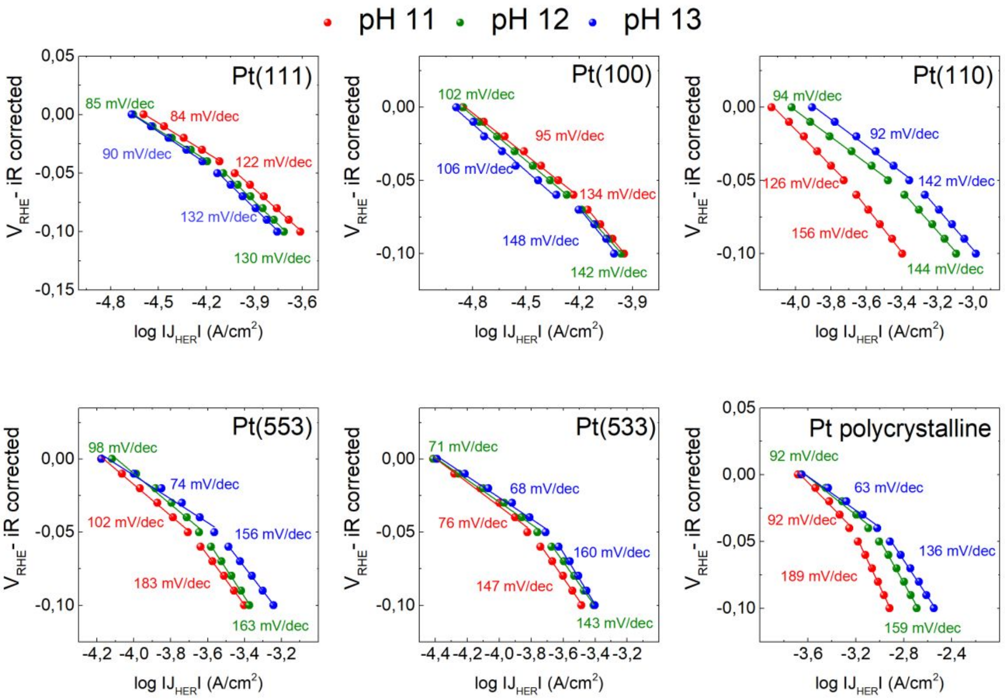

Fig. S5. *Tafel data for HER (potential vs log of current density) as obtained from chronoamperometry measurements at different Pt surfaces in 0.1 M NaOH (pH 13), 0.01 M NaOH +0.09 M NaClO4 (pH 12) and 0.001 M NaOH + 0.099 M NaClO4 (pH 11) recorded at 20 mV potential steps. All the measurements on Pt single crystals were done in stationary hanging meniscus configuration and the measurement at the polycrystalline Pt rotating disk electrode was done at 2500 rpm. The Tafel slopes (mVdec^{-1}) at each pH are indicated next to the plots. Note that the Tafel slope is not constant, though at the most negative potentials is consistently close to 120 mV/dec.*

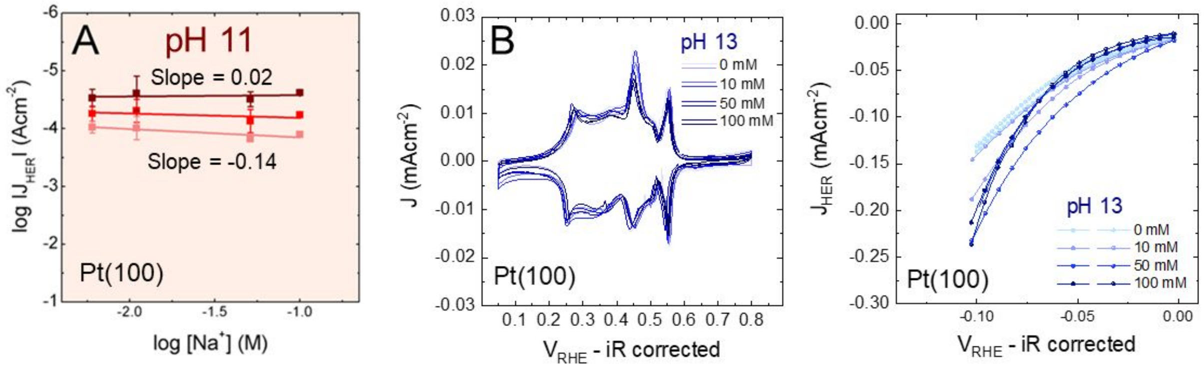

Fig. S6. *Logarithm of HER current density on Pt(100) plotted against the logarithm of [Na^+] ion concentration at **(A)** pH 11. The corresponding slopes (reaction orders) are indicated next to the plots, where the slope at the bottom corresponds to the applied potential of -0.01 V_{RHE} (iR corrected) and the slope at the top corresponds to the applied potential of -0.1 V_{RHE} (iR corrected). (B) Blank CVs of Pt(100) in pH=13 and HER current between -0.1 V and 0 V vs RHE for Pt(100) electrodes (each measured after another annealing treatment) and with different background concentrations of $NaClO_4$. The relatively poor trend in the data is presumably to the different number of defects in Pt(100) after each annealing treatment, leading to different activities.*

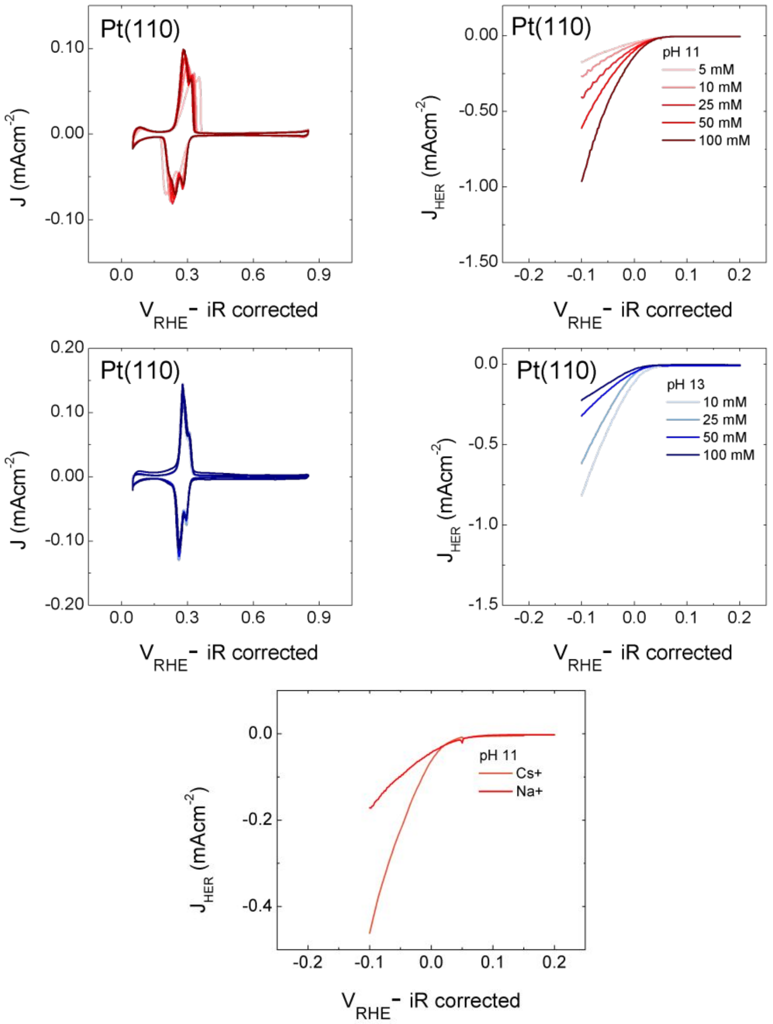

Fig. S7. (upper row) *Blank CV (left) and HER activity measurement (right) on Pt(110) at pH 11 at different concentrations of* $NaClO_4$ *(increasing concentration of* $NaClO_4$ *in going from light to dark data points).* ***(middle row)*** *Blank CV (left) and HER activity measurement (right) on Pt(110) at pH 13 at different concentrations of* $NaClO_4$ *(increasing concentration of* $NaClO_4$ *in going from light to dark data points).* ***(lower row)*** *Effect of* Cs^+ *vs.* Na^+ *on Pt(110) in pH=11 NaOH + 5 mM of either* $NaClO_4$ *or* $CsClO_4$*.*

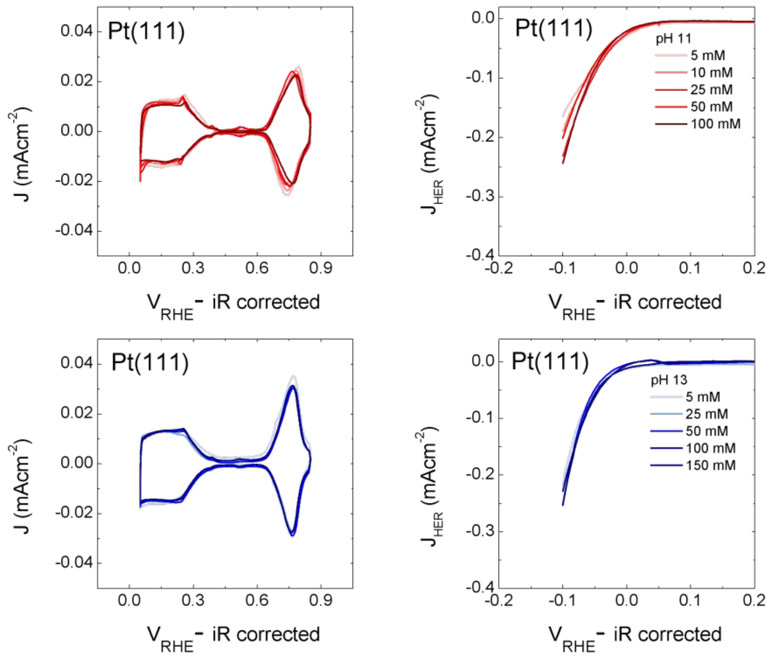

Fig. S8. (A) *Blank CV (left) and HER activity measurement (right) on Pt(111) at pH 11 at different concentrations of* $NaClO_4$ *(increasing concentration of* $NaClO_4$ *in going from light to dark data points).* ***(B)*** *Blank CV (left) and HER activity measurement (right) on Pt(111) at pH 13 at different concentrations of* $NaClO_4$ *(increasing concentration of* $NaClO_4$ *in going from light to dark data points).*

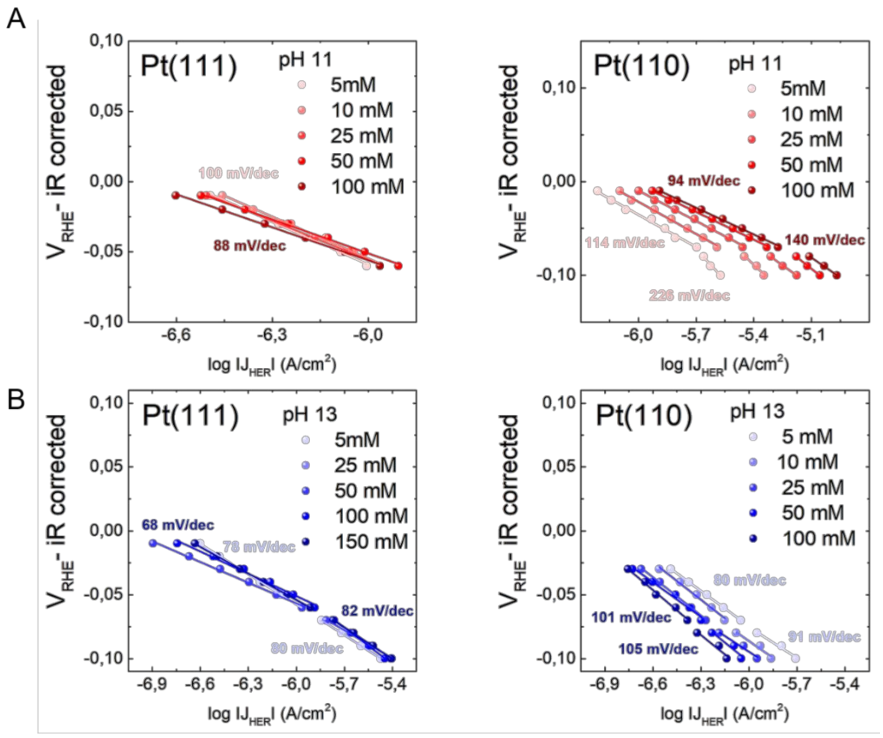

Fig. S9. *Tafel data for HER (potential vs log of current density) as obtained from chronoamperometry measurements at Pt(111) (left) and Pt(110) (right) at different concentrations of* $NaClO_4$ *(increasing concentration of* $NaClO_4$ *in going from light to dark data points) in* ***(A)*** *pH 11 and* ***(B)*** *pH 13 electrolytes.*

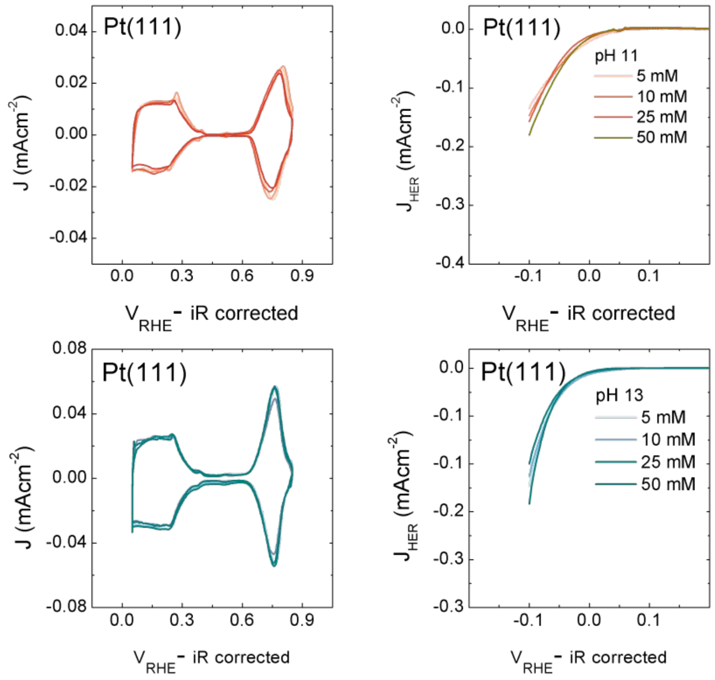

Fig. S10. (A) *Blank CV (left) and HER activity measurement (right) on Pt(111) at pH 11 at different concentrations of $CsClO_4$ (increasing concentration of $CsClO_4$ in going from light to dark data points).* ***(B)*** *Blank CV (left) and HER activity measurement (right) on Pt(111) at pH 13 at different concentrations of $CsClO_4$ (increasing concentration of $CsClO_4$ in going from light to dark data points).*

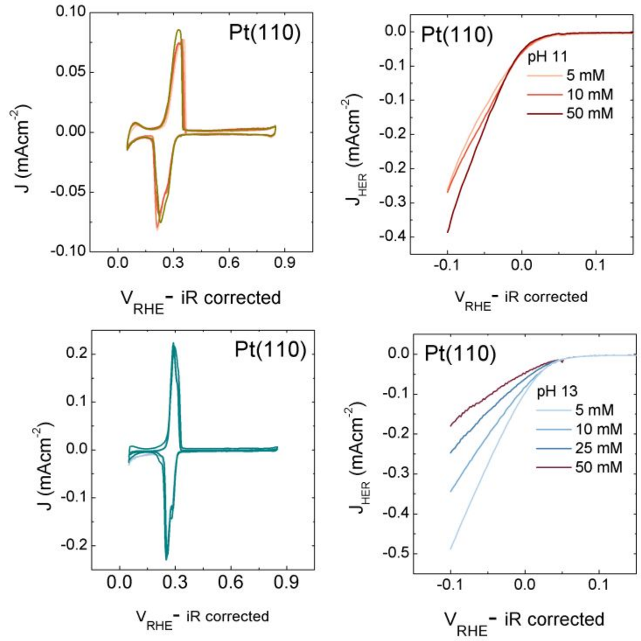

Fig. S11. (A) *Blank CV (left) and HER activity measurement (right) on Pt(110) at pH 11 at different concentrations of $CsClO_4$ (increasing concentration of $CsClO_4$ in going from light to dark data points).* ***(B)*** *Blank CV (left) and HER activity measurement (right) on Pt(110) at pH 13 at different concentrations of $CsClO_4$ (increasing concentration of $CsClO_4$ in going from light to dark data points).*

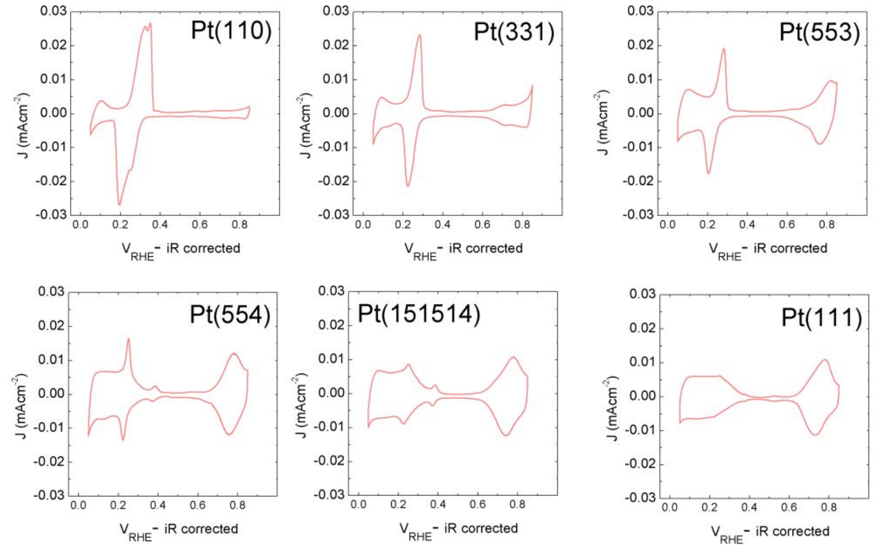

Fig. S12. *Blank CVs of Pt(110), Pt(331), Pt(553), Pt(554), Pt(151514) and Pt(111) as recorded in 0.001 M NaOH (pH 11) plus 0 mM $NaClO_4$ at a scan rate of 10 mVs^{-1}. For the preparation of all the single crystals the procedure outlined in Materials and Methods subsection 2 was followed.*

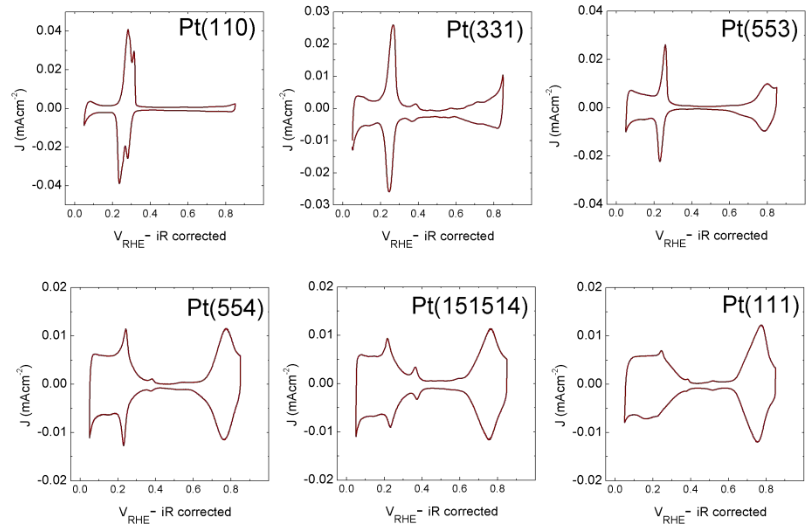

Fig. S13. *Blank CVs of Pt(110), Pt(331), Pt(553), Pt(554), Pt(151514) and Pt(111) as recorded in 0.001 M NaOH (pH 11) plus 50 mM $NaClO_4$ at a scan rate of 10 mVs^{-1}. For the preparation of all the single crystals the procedure outlined in Materials and Methods subsection 2 was followed.*

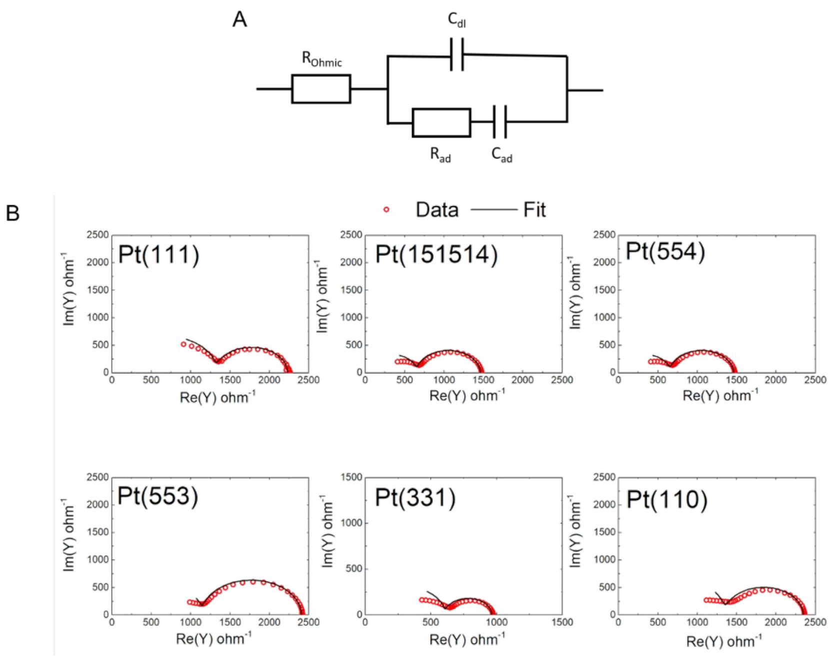

Fig. S14. (A) *EEC1 used to fit the data in the H_{upd} region; it features R_{Ohmic} which represents the solution resistance, C_{dl} which represents the double layer capacitance and R_{ad} , C_{ad} which together represent the kinetics of H adsorption at the surface.(10)* ***(B)*** *Nyquist admittance plots with the corresponding fits obtained with EEC1 at 0.04 V_{RHE} at different Pt single crystals in 0.001 M NaOH (pH 11).*

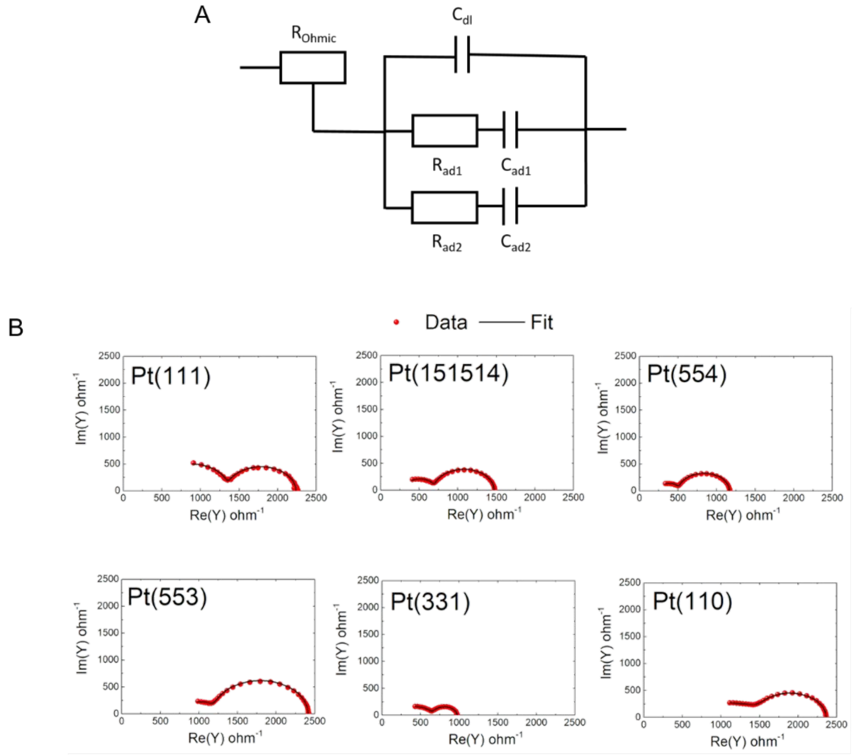

Fig. S15. (A) *EEC2 which is a modification of EEC1and features an additional branch that takes into account any co-adsorption phenomenon in the H_{upd} region;(9) it features R_{Ohmic} which represents the solution resistance, C_{dl} which represents the double layer capacitance, R_{ad1} , C_{ad1} which together represent the kinetics of H adsorption at the surface and R_{ad2} , C_{ad2} which together represent the kinetics of co-adsorption of a secondary species at the surface.(10)* ***(B)*** *Nyquist admittance plots with the corresponding fits obtained with EEC2 at 0.04 V_{RHE} at different Pt single crystals in 0.001 M NaOH (pH 11).*

Pt(S)-[n(111) x (110)]	C_{dl} (µFcm^{-2}) EEC1	C_{dl} (µFcm^{-2}) EEC2
**Pt(110)**	28	26
**Pt(331)**	23	22.3
**Pt(553)**	21.6	22
**Pt(554)**	21	20.7
**Pt(151514)**	20.3	19.9
**Pt(111)**	17.9	16.7

Table S1. *The values of specific double layer capacitance (C_{dl}; µFcm^{-2}) as derived from EEC1 (shown in Fig. S14) and EEC2 (as shown in Fig. S15) at 0.04 V_{RHE} on different Pt single crystals in 0.001 M NaOH (pH 11).*

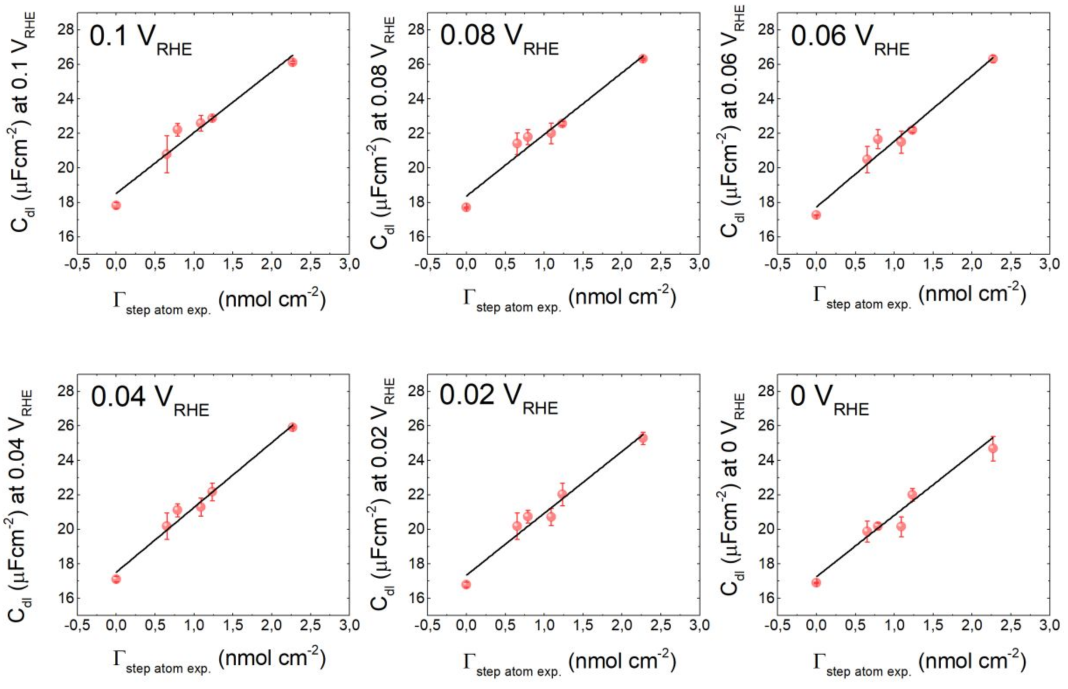

Fig. S16. *Specific double layer capacitance (C_{dl}; µFcm^{-2}) as derived by using the equivalent electric circuit shown in Supplementary Fig. S15 on different Pt single crystals at different potentials (vs RHE) in 0.001 M NaOH (pH 11) as a function of experimentally derived step density ($\Gamma_{step\ atom\ exp.}$; nmol cm^{-2}). The experimental step density ($\Gamma_{step\ atom\ exp.}$) was calculated by integrating the charge for the hydrogen adsorption peak as obtained from the bank cyclic voltammograms shown in Fig. S11.*

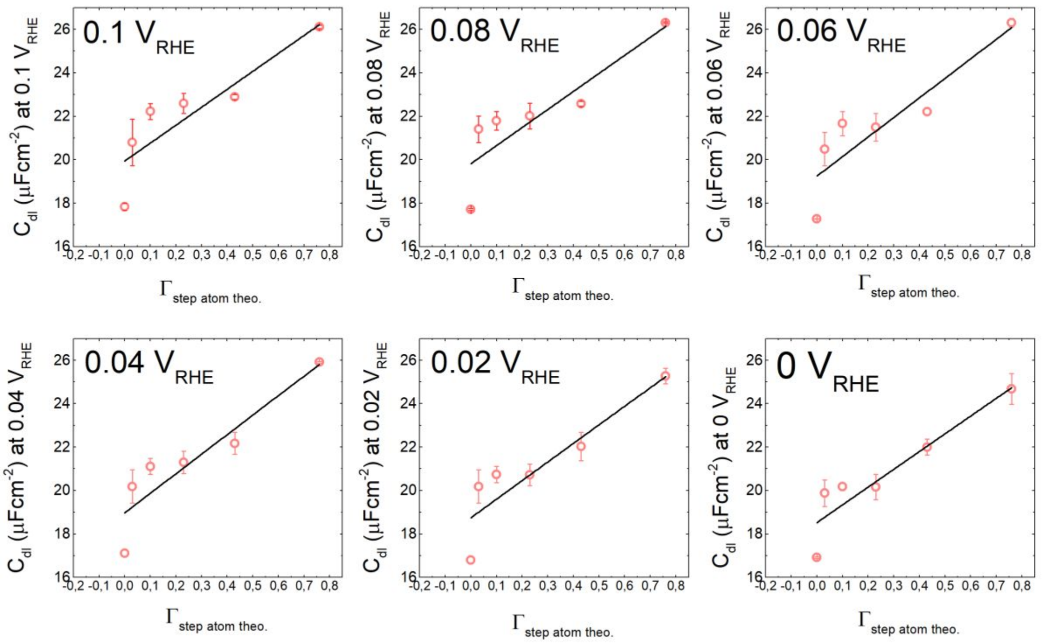

Fig. S17. *Specific double layer capacitance (C_{dl}; μFcm^{-2}) as derived by using the equivalent electric circuit shown in Supplementary Fig. S15 on different Pt single crystals at different potentials (vs RHE) in 0.001 M NaOH (pH 11) as a function of theoretical step density ($\Gamma_{step\ atom\ theo.}$) which was calculated by using the procedure discussed in Supplementary Text subsection 1.*

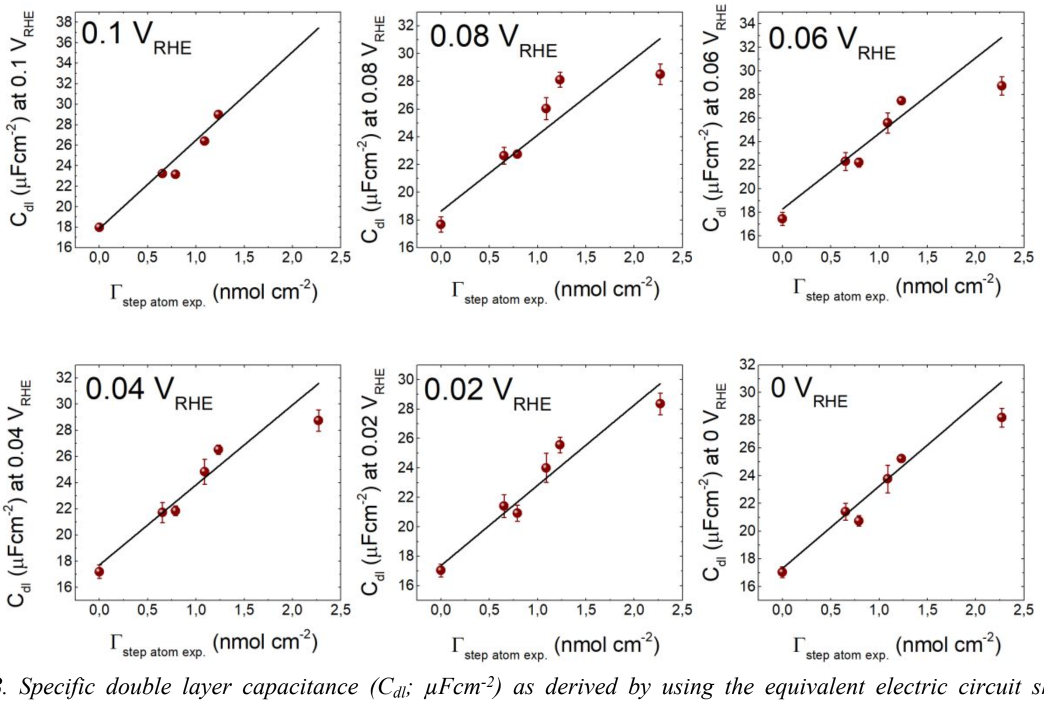

Fig. S18*. Specific double layer capacitance (C_{dl}; μFcm^{-2}) as derived by using the equivalent electric circuit shown in Supplementary Fig. S15 on different Pt single crystals at different potentials (vs RHE) in 0.001 M NaOH (pH 11) plus 50 mM $NaClO_4$ as a function of experimentally derived step density ($\Gamma_{step\ atom\ exp.}$; $nmolcm^{-2}$). The experimental step density ($\Gamma_{step\ atom\ exp.}$) was calculated by integrating the charge for the hydrogen adsorption peak as obtained from the bank cyclic voltammograms shown in Fig. S11.*

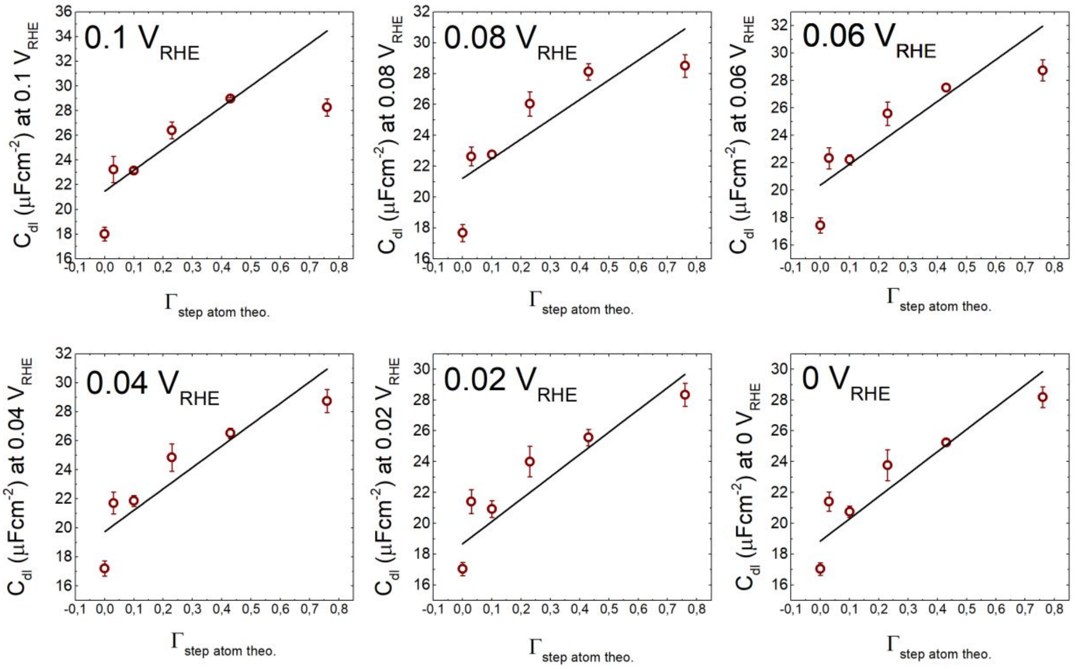

Fig. S19. *Specific double layer capacitance (C_{dl}; μFcm^{-2}) as derived by using the equivalent electric circuit shown in Supplementary Fig. S15 on different Pt single crystals at different potentials (vs RHE) in 0.001 M NaOH (pH 11) plus 50 mM $NaClO_4$ as a function of theoretical step density ($\Gamma_{step\ atom\ theo.}$) which was calculated by using the procedure discussed in Supplementary Text subsection 1.*

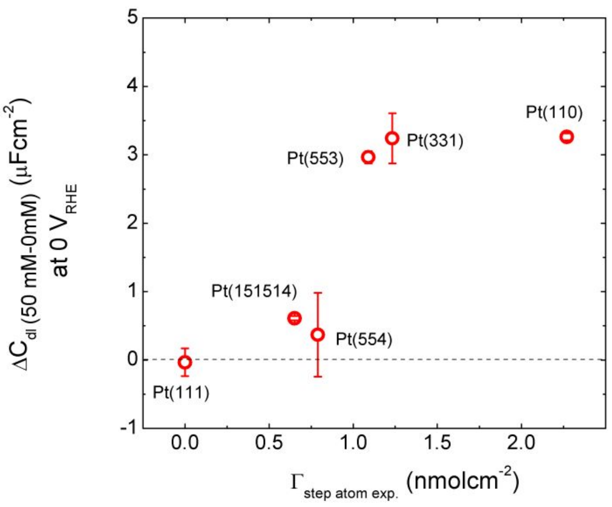

Fig. S20. *Delta of specific double layer capacitance (ΔC_{dl}; μFcm^{-2}) at pH 11 in 50 mM and 0 mM $NaClO_4$ containing electrolyte as derived by using the equivalent electric circuit shown in Supplementary Fig. S16 on different Pt single crystals at 0 V (vs RHE) as a function of experimental step density ($\Gamma_{step\ atom\ exp.}$; nmolcm^{-2}). The experimental step density ($\Gamma_{step\ atom\ exp.}$) was calculated by integrating the charge for the hydrogen adsorption peak as obtained from the bank cyclic voltammograms shown in Fig. S11.*

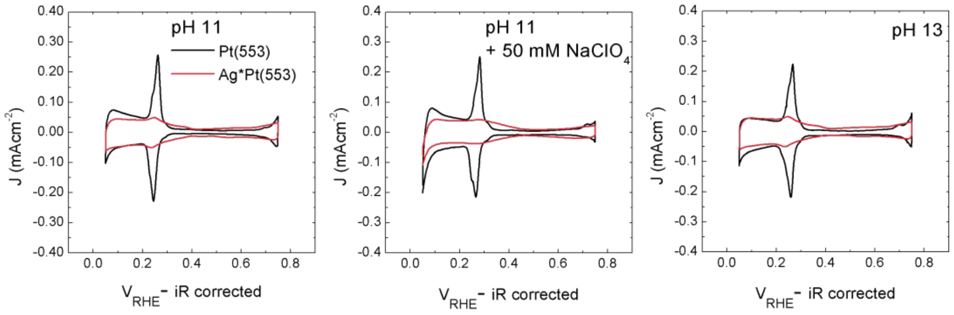

Fig. S21. *Blank Pt(553) (black) and Ag modified Pt(553) (pink) in 0.001 M NaOH (left), 0.001 M NaOH plus 50 mM $NaClO_4$ (middle) and 0.1 M NaOH (right).*

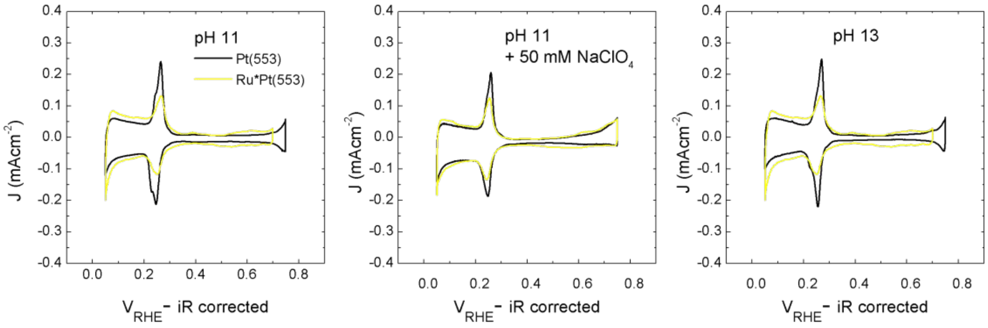

Fig. S22. *Blank CV of bare Pt(553) (black) and Ru modified Pt(553) (yellow) 0.001 M NaOH (left), 0.001 M NaOH plus 50 mM $NaClO_4$ (middle) and 0.1 M NaOH (right).*

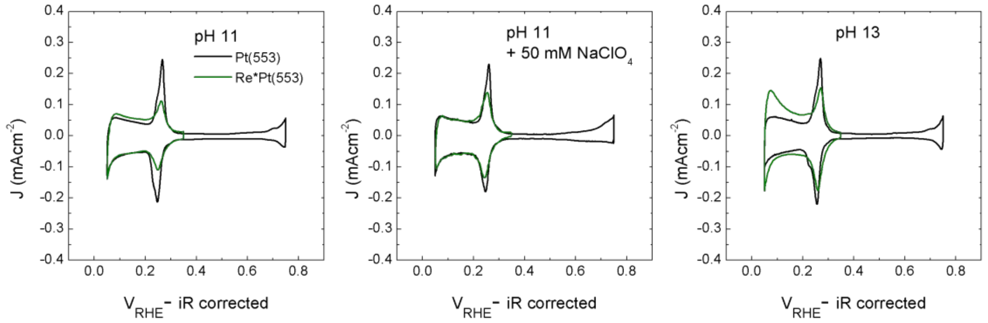

Fig. S23. *Blank CV of bare Pt(553) (black) and Re modified Pt(553) (green) 0.001 M NaOH (left), 0.001 M NaOH plus 50 mM $NaClO_4$ (middle) and 0.1 M NaOH (right). For Re modified Pt(553) potentials more anodic than 0.35 V_{RHE} were avoided to prevent the oxidation of the adatoms.*

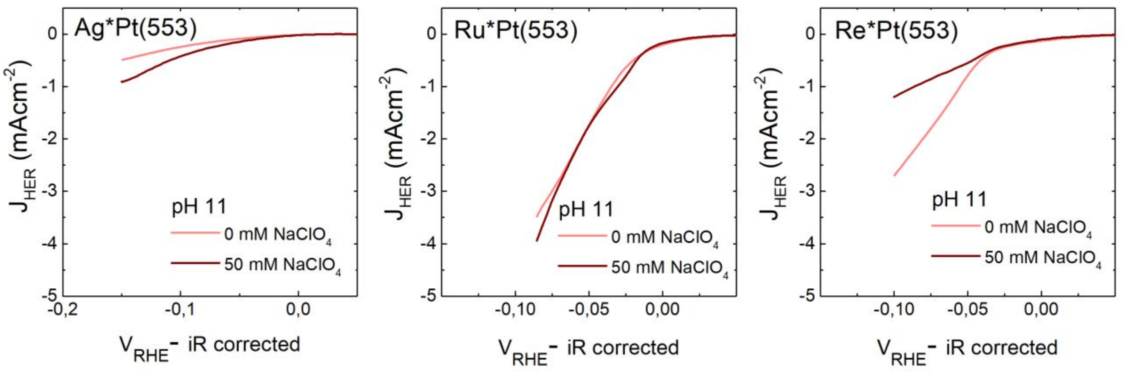

Fig. S24. *CVs showing HER activity trends on different adatom modified Pt(553) surfaces in 0.001 M NaOH plus 0 mM $NaClO_4$ (light red) and in 0.001 M NaOH plus 50 mM $NaClO_4$ (dark red). In going from left to right, the adatom oxophylicity increases. Scan rate: 10 mVs^{-1}.*

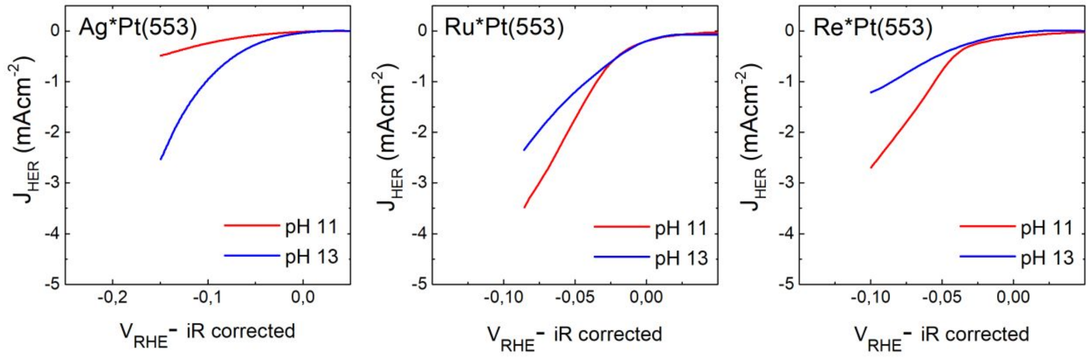

Fig. S25. *CVs showing HER activity trends on different adatom modified Pt(553) surfaces at pH 11 (red) and pH 13 (blue). In going from left to right, the adatom oxophilicity increases. Scan rate: 10 mVs^{-1}.*

Supplementary References:

1. L. A. Kibler, A. Cuesta, M. Kleinert, D. M. Kolb, In-situ STM characterisation of the surface morphology of platinum single crystal electrodes as a function of their preparation. *Journal of Electroanalytical Chemistry* **484**, 73-82 (2000).
2. P. Rodríguez, G. García, E. Herrero, J. M. Feliu, M. T. M. Koper, Effect of the Surface Structure of Pt(100) and Pt(110) on the Oxidation of Carbon Monoxide in Alkaline Solution: an FTIR and Electrochemical Study. *Electrocatalysis* **2**, 242 (2011).
3. G. A. Attard, A. Brew, Cyclic voltammetry and oxygen reduction activity of the Pt{110}-(1×1) surface. *Journal of Electroanalytical Chemistry* **747**, 123-129 (2015).
4. M. Łukaszewski, M. Soszko, A. Czerwiński, *Electrochemical Methods of Real Surface Area Determination of Noble Metal Electrodes – an Overview.* (2016), vol. 11, pp. 4442-4469.
5. S. Xue, B. Garlyyev, A. Auer, J. Kunze-Liebhäuser, and A. S. Bandarenka, How the Nature of the Alkali Metal Cations Influences the Double-Layer Capacitance of Cu, Au, and Pt Single-Crystal Electrodes. *The Journal of Physical Chemistry C* 124, 12442-12447 (2020)

6. I. T. McCrum, M. T. M. Koper, The role of adsorbed hydroxide in hydrogen evolution reaction kinetics on modified platinum. *Nature Energy* 5, 891-899 (2020).
7. H. Massong, H. Wang, G. Samjeské, H. Baltruschat, The co-catalytic effect of Sn, Ru and Mo decorating steps of Pt(111) vicinal electrode surfaces on the oxidation of CO. *Electrochimica Acta* 46, 701-707 (2001).
8. J. Clavilier, K. El Achi, A. Rodes, In situ probing of step and terrace sites on Pt(S)-[n(111) × (111)] electrodes. *Chemical Physics* 141, 1-14 (1990).
9. C. J. Bondue, F. Calle-Vallejo, M. C. Figueiredo, M. T. M. Koper, Structural principles to steer the selectivity of the electrocatalytic reduction of aliphatic ketones on platinum. *Nature Catalysis* 2, 243-250 (2019).
10. S. Morin, H. Dumont, B. E. Conway, Evaluation of the effect of two-dimensional geometry of pt single-crystal faces on the kinetics of upd of h using impedance spectroscopy. *Journal of Electroanalytical Chemistry* 412, 39-52 (1996).
11. K. J. P. Schouten, M. J. T. C. van der Niet, M. T. M. Koper, Impedance spectroscopy of H and OH adsorption on stepped single-crystal platinum electrodes in alkaline and acidic media. *Physical Chemistry Chemical Physics* 12, 15217-15224 (2010).
12. A. J. Bard, L. R. Faulkner, *Electrochemical methods : fundamentals and applications* (Wiley, New York, 1980).
13. K. Ojha, K. Doblhoff-Dier, M. T. M. Koper, Double-layer structure of the Pt(111)–aqueous electrolyte interface. *Proceedings of the National Academy of Sciences* 119, e2116016119 (2022).
14. M. J. T. C. van der Niet, N. Garcia-Araez, J. Hernández, J. M. Feliu, M. T. M. Koper, Water dissociation on well-defined platinum surfaces: The electrochemical perspective. *Catalysis Today* 202, 105-113 (2013).
15. I. T. McCrum, M. J. Janik, pH and Alkali Cation Effects on the Pt Cyclic Voltammogram Explained Using Density Functional Theory. *The Journal of Physical Chemistry C* 120, 457-471 (2016).
16. I. T. McCrum, M. J. Janik, First Principles Simulations of Cyclic Voltammograms on Stepped Pt(553) and Pt(533) Electrode Surfaces. *ChemElectroChem* 3, 1609-1617 (2016).
17. G. García, M. T. M. Koper, Mechanism of electro-oxidation of carbon monoxide on stepped platinum electrodes in alkaline media: a chronoamperometric and kinetic modeling study. *Physical Chemistry Chemical Physics* 11, 11437-11446 (2009).
